# Supplementary material for: Photoactivation of silicon rhodamines via a light-induced protonation
Source: Nat Commun. 2019 Oct 8;10:4580. doi: 10.1038/s41467-019-12480-3 (PMC6783549; doi:10.1038/s41467-019-12480-3)
Supplement: Supplementary file 1 — Supplementary Information [file 41467_2019_12480_MOESM1_ESM.pdf]

Supplementary Information

Photoactivation of silicon rhodamines via a light-induced protonation

Frei et al.

Supplementary Figures

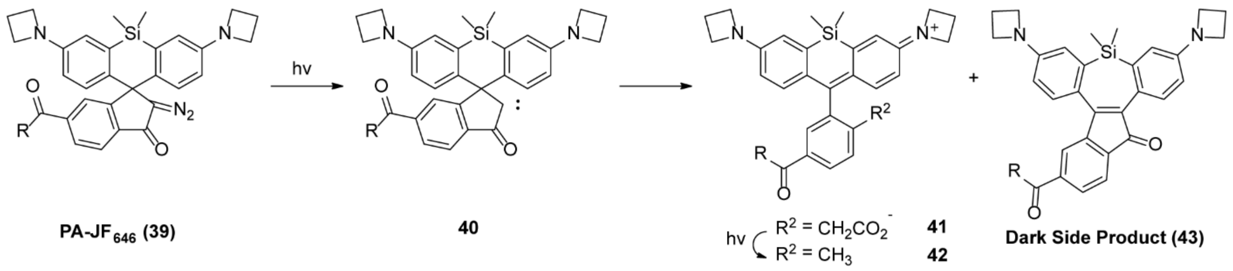

Supplementary Figure 1. Photoactivation of PA-JF$_{646}$. Showing the reactive carbene intermediate **40** along with the two fluorescent products and the dark side-product formed.

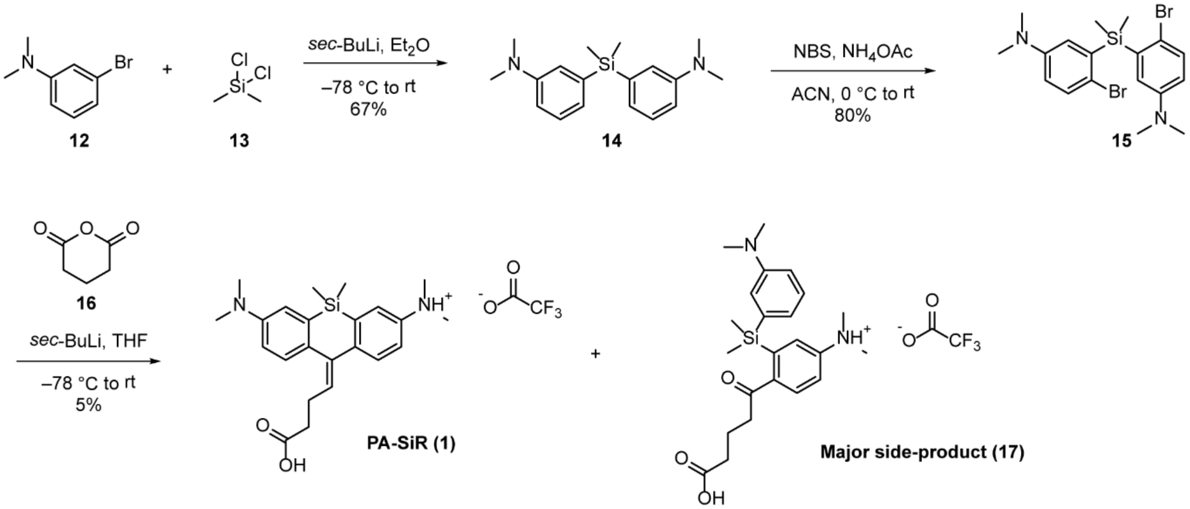

Supplementary Figure 2. Synthesis of PA-SiR (**1**). The products of the first two reaction steps could both be isolated with good yields, 67% and 80% respectively. However, the final reaction step, closure of the xanthene core, proceeds with substantially lower yield. This is mainly due to the formation of **17** as a major side-product, which is formed upon abstraction of an alpha-proton of glutaric anhydride. This reaction pathway is disfavored when using substrates with lower acidity. This is confirmed by the higher yields observed in the synthesis of compounds **4** and **20.** Initial attempts to increase yields of the synthesis of PA-SiR via Grubbs metathesis of **4** or Wittig reaction with an analogues ketone were not successful (data not shown).

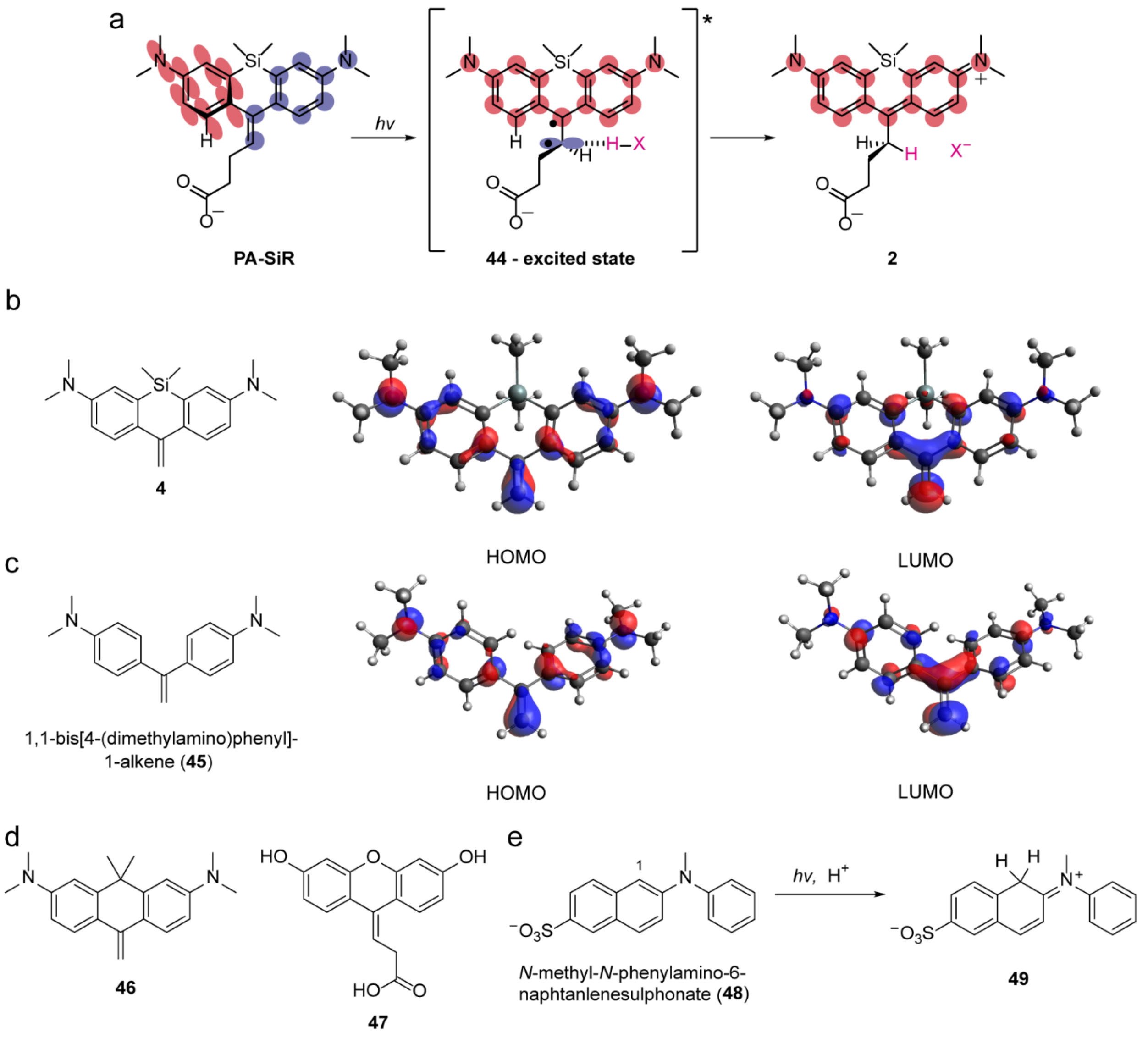

Supplementary Figure 3. Proposed reaction mechanism for photoactivation of PA-SiR and related data. (**a**) Possible mechanism for photoactivation of PA-SiR. The twisted structure of compound **4** reduces unfavorable steric interactions (Fig. 1b, and presumably PA-SiR). Moreover, it indicates two separate, cross-conjugated π-systems: one comprising an aniline ring and the double bond (colored in blue) and the other comprising just the aniline ring (colored in red). Upon irradiation a diradical (excited state **44**) is formed. The two π-systems become conjugated and unfavorable steric interactions are released by bond rotation around the bond connecting C9 and C10. The alkyl radical on C10 can be stabilized through H-bonding by the solvent. Electron transfer from the aromatic system to the alkyl radical followed by protonation yields compound **2**. (**b**) Structure of model PA-SiR **4** together with calculated HOMO and LUMO (B3LYP/6-31G(d), only contributions bigger than 0.05 are shown). According to these calculations, the LUMO receives dominant contributions from the exocyclic double bond whereas the HOMO locates

to the anilines. This indicates that a light-induced HOMO-LUMO transition would lead to an intramolecular charge transfer. (**c**) Structure along with calculated HOMO and LUMO of model compound 1,1-bis[4-(dimethyl-amino)phenyl]-1-alkene (**45**). Substituted 1,1-diphenylethenes are known to exhibit twisted intramolecular charge transfer states in polar solvents and are therefore comparable to PA-SiRs[1]. The most closely related 1,1-bis[4-(dimethyl-amino)phenyl]-1-alkene shown here is reported to convert to a colored species upon irradiation, but the phenomenon was not further investigated[2]. Its LUMO receives dominant contributions from the extracyclic double bond, whereas the HOMO is localized on the periphery of the molecule similarly to PA-SiR **4**. (**d**) Structures of the reported olefinic rhodamine derivatives: carbopyronine **46**[3] and the olefinic fluorescein **47**[4]. They were not reported to be photoactivatable. (**e**) Protonation of *N*-methyl-*N*-phenylamino-6-naphtanlenesulphonate (**48**) to protonated **49**[5,6].

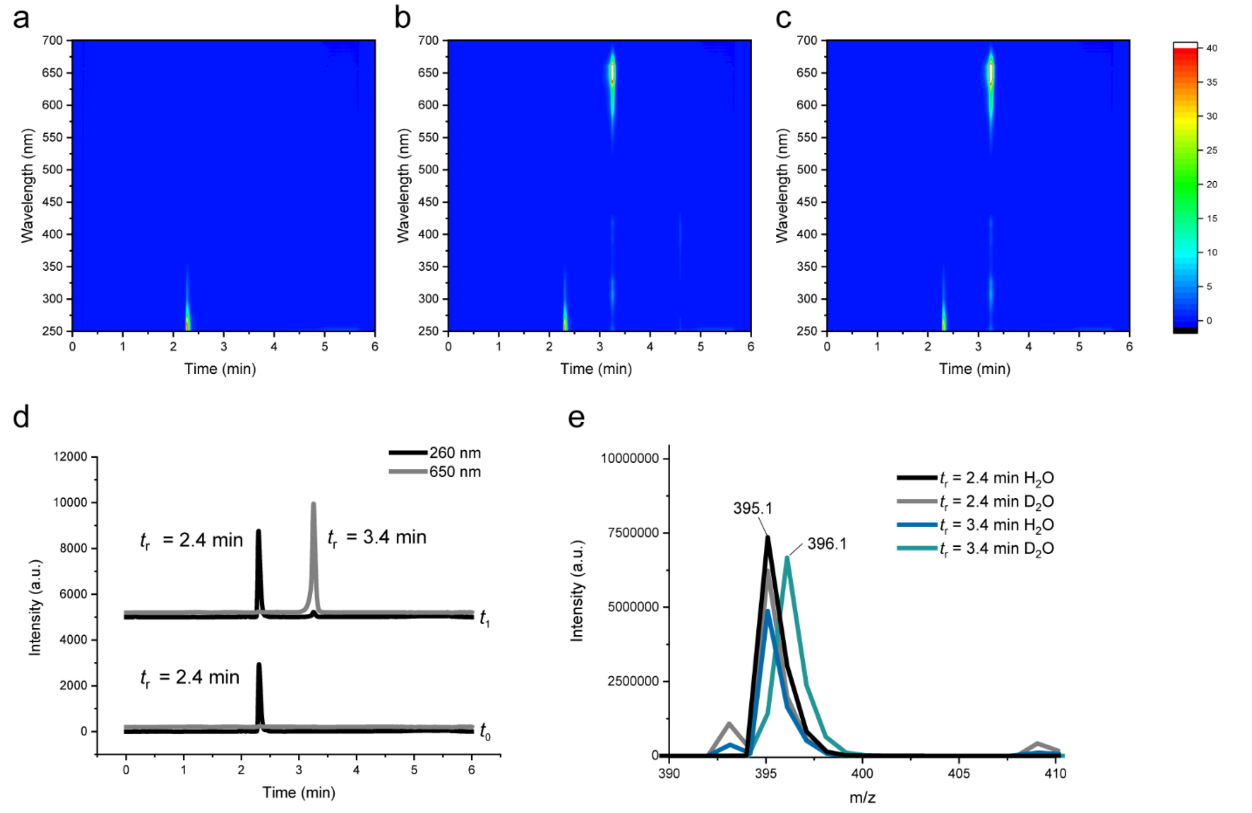

Supplementary Figure 4. LC-MS analysis of the photoactivation. (**a**-**c**) LC-MS 3D contour plots of aqueous PA-SiR solution before **b**, directly after **c** and further 15 min in the dark after activation ensuring complete decay and formation of **3** (iii). It is not possible to observe **3** under the acidic LC-MS conditions. Only PA-SiR and SiR **2** are observed (peaks at 2.4 min and 3.4 min). A minor impurity formed during the reaction is visible at 4.8 min (*m/z* = 325.1, 366.1). (**d**) Isotope experiment: LC-MS traces of aqueous (D_2O) PA-SiR solutions before (t_0) and after activation with UV light for 30 s (t_1) at 260 nm and 650 nm. Retention times (t_r) are given. (**e**) Relevant *m/z* signals of the two peaks in both H_2O and D_2O experiments. The peak at 2.4 min shows the same *m/z* = 395.1 ratio corresponding to PA-SiR $[M+H]^+$ in both solvents (H_2O and D_2O). The peak at 3.4 min in D_2O has a *m/z* = 396.1 corresponding to deuterated SiR **2** $[M]^+$ confirming protonation/deuteration.

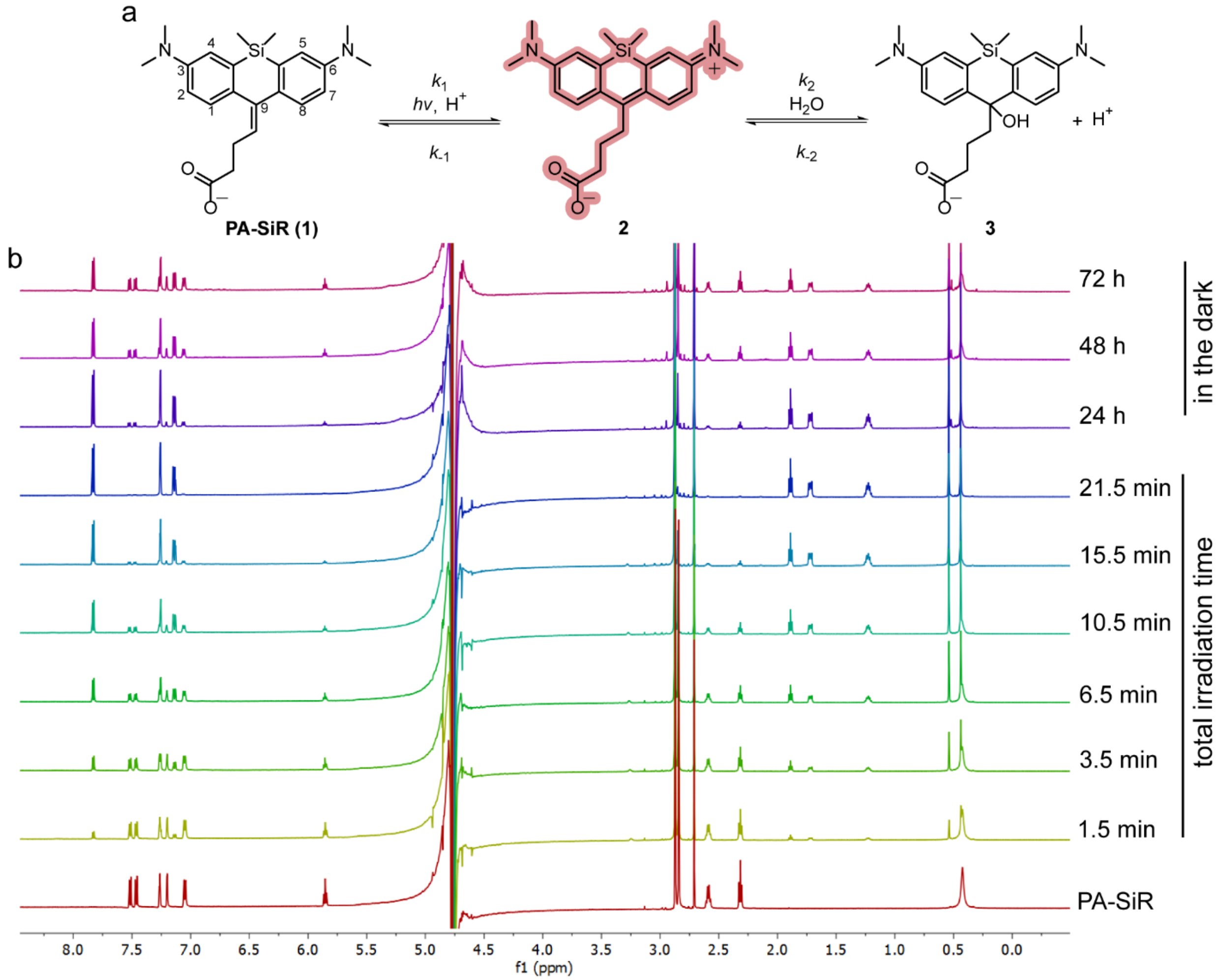

Supplementary Figure 5. ^{1}H nuclear magnetic resonance (NMR) photoactivation experiments. (**a**) Equilibrium system of PA-SiR, SiR **2** and **3**. (**b**) ^{1}H NMR spectra of PA-SiR (2.0 mM in PBS) before, during and after UV irradiation. Irradiation times are given as total irradiation times. Assignment of the pure spectra of PA-SiR and at 21.5 min of **3**: ^{1}H NMR (PA-SiR, 600 MHz, H_2O + D_2O): δ 7.52 (d, J = 8.5 Hz, 1H; CH_{ar}), 7.46 (d, J = 8.5 Hz, 1H; CH_{ar}), 7.26 (d, J = 2.8 Hz, 1H; CH_{ar}), 7.20 (d, J = 2.8 Hz, 1H; CH_{ar}), 7.06 (d, J = 1.7 Hz, 1H; CH_{ar}), 7.04 (d, J = 1.6 Hz, 1H; CH_{ar}), 5.85 (t, J = 7.4 Hz, 1H; C*H*), 2.87 (s, 6H; N*Me*), 2.84 (s, 6H; N*Me*), 2.71 (s, 2H; DMSO reference), 2.59 (q, J = 7.5 Hz, 2H; CH_2), 2.32 (t, J = 7.5 Hz, 2H; CH_2), 0.43 (s, 6H; SiMe_2); and ^{1}H NMR (21.5 min **3**, 600 MHz, H_2O + D_2O): δ 7.83 (d, J = 8.8 Hz, 2H; CH_{ar}), 7.26 (d, J = 2.8 Hz, 2H; CH_{ar}), 7.14 (dd, J = 8.9, 2.8 Hz, 2H; CH_{ar}), 2.87 (s, 12H; NMe_2), 2.71 (s, 3H; DMSO reference), 1.89 (t, J = 7.4 Hz, 2H; CH_2), 1.66 – 1.80 (m, 2H; CH_2), 1.12 – 1.29 (m, 2H; CH_2), 0.54 (s, 3H; Si*Me*), 0.44 (s, 3H; Si*Me*). The signal around 4.7 ppm corresponds to the residual H_2O signal that was not suppressed. **2** is not visible under the chosen conditions pH= 7–8. This experiments shows that the photoactivation reaction is reversible over a longer time scale, where either k_{-1} or a third process k_3 becomes important.

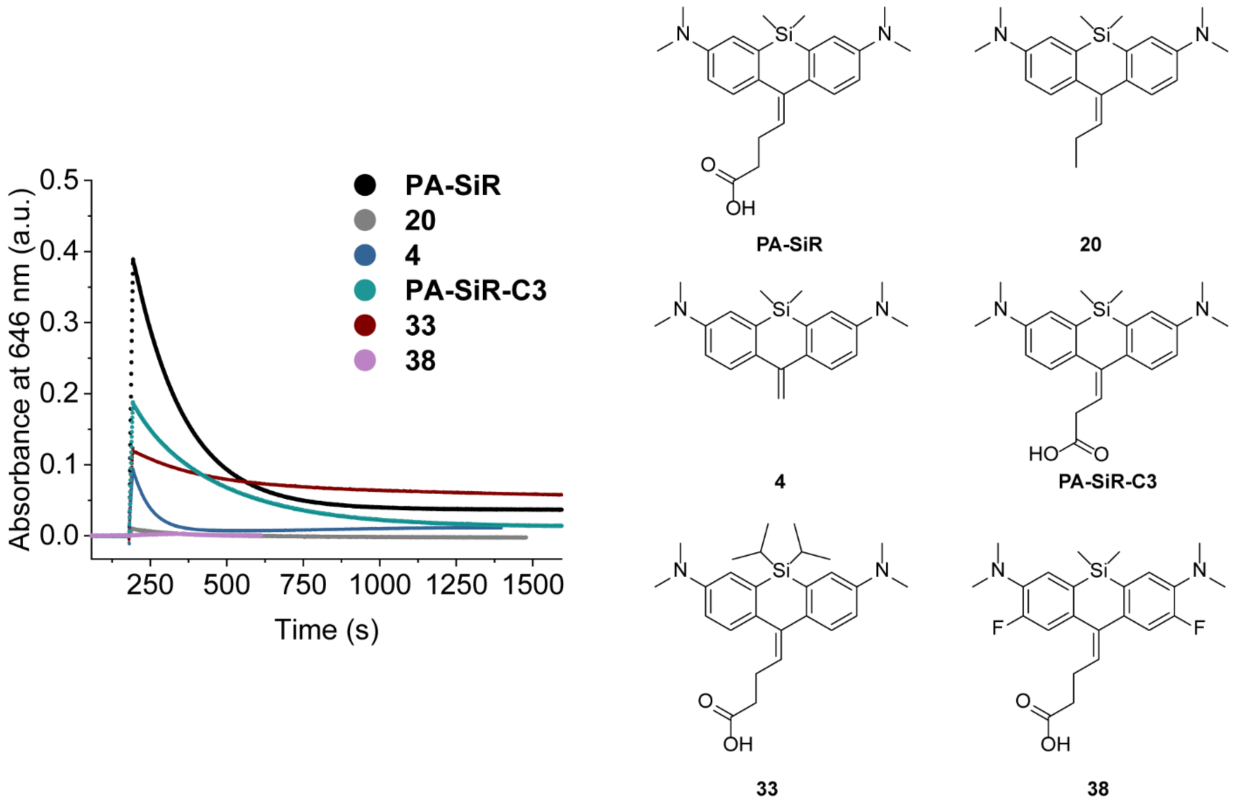

Supplementary Figure 6. Time dependent absorbance measurements for different PA-SiR analogues. Measurements were carried out at 10 µm in PBS at 646 nm. Activation was performed for 12 s after 3 min. These measurements let us conclude that the disappearance of the absorbance peak at 646 nm of photoactivated PA-SiR analogues is due to a reaction with a water molecule and not spirolactonization, as the removal of the carboxylic acid group of PA-SiR, leading to compounds **20** and **4**, did not prevent the disappearance of the far-red absorbance. However, we cannot exclude that spirolactonization contributes to the disappearance of the far-red absorbance observed for PA-SiR, PA-SiR-C3, **33** and **38**. Upon structural variation, both faster and slower reaction rates k_2 and different equilibrium positions relative to PA-SiR were found. For instance, fluorination at the aromatic core lead to PA-SiR derivatives, which only showed a change in absorbance after prolonged UV irradiation. Bulky ^{i}Pr groups on Si drastically decreased the reaction rates of the nucleophilic attack k_2.. Fitted decay parameters are given in Supplementary Table 7.

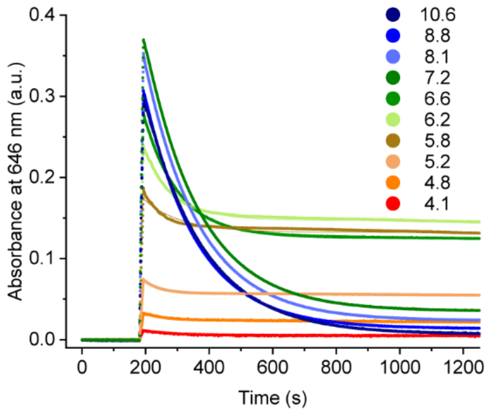

Supplementary Figure 7. Influence of different pH values on the equilibrium system. Absorbance measurements at 646 nm over time for PA-SiR solutions at 10 µM in PBS with pH values ranging from 4.1–10.6. These measurements were used to extract the normalized A_{max} and A_{eq} (normalized relative to A_{max}) values presented in Fig. 1e. All data were fitted with a mono-exponential decay function. The curves obtained at pH values between 6.6 and 4.8 show a bi-exponential decay, indicating the presence of a second component. It is likely that the reaction k_{-1} becomes relevant under these conditions. Therefore, considering exclusively the second equilibrium (represented by k_2 and k_{-2}) for the fits is only an approximation.

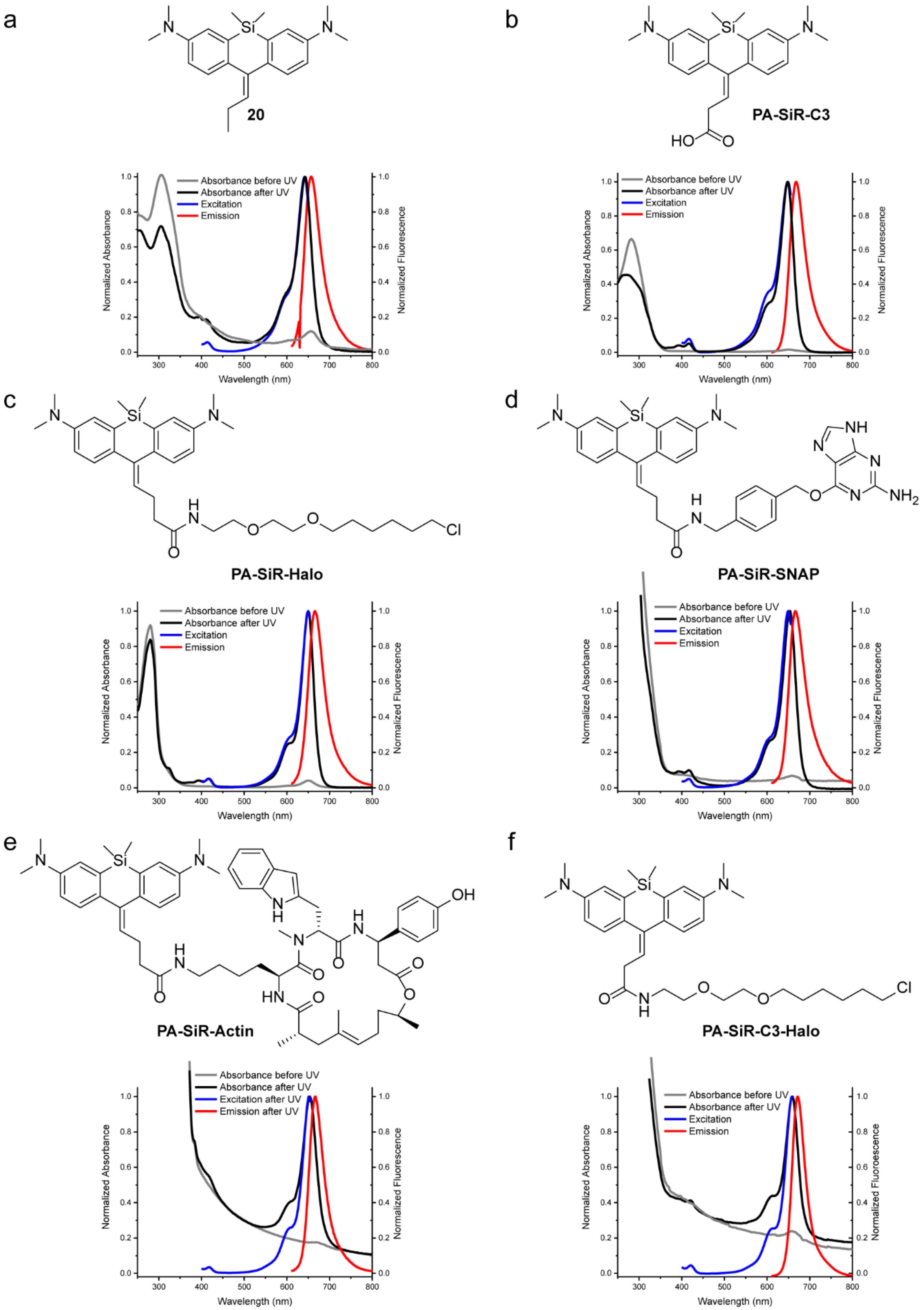
a
20
b
PA-SiR-C3
c
PA-SiR-Halo
d
PA-SiR-SNAP
e
PA-SiR-Actin
f
PA-SiR-C3-Halo
Absorbance before UV
Absorbance after UV
Excitation
Emission
Excitation after UV
Emission after UV
Normalized Absorbance
Normalized Fluorescence
Wavelength (nm)

Supplementary Figure 8. Structures and absorption, excitation and emission spectra of PA-SiR derivatives. (**a**) **20**, (**b**) PA-SiR-C3, (**c**) PA-SiR-Halo conjugated to HaloTag, (**d**) PA-SiR-SNAP conjugated to SNAP-tag, (**e**) PA-SiR-Actin in the presence of F-actin, (**f**) PA-SiR-C3-Halo conjugated to HaloTag. Absorption spectra were measured before and after UV irradiation. All spectra were normalized to their maximum between 600 and 700 nm except the absorbance spectra before UV irradiation, which was normalized to the same factor as the spectra recorded after UV irradiation.

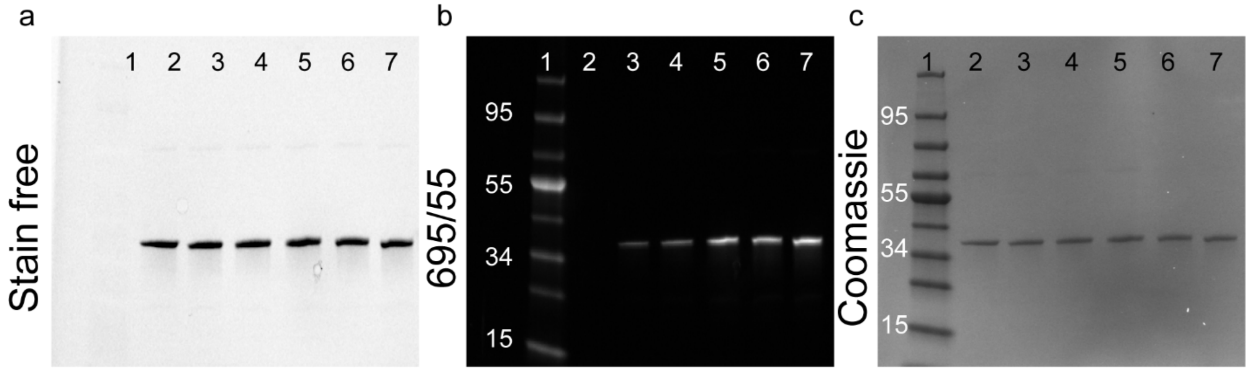

Supplementary Figure 9. Analysis of labeling of HaloTag with PA-SiR-Halo using PAGE. (**a**) Stain free gel imaged after UV-activation showing the fluorescence of tryptophan reacted with a proprietary component of the stain free gel. (**b**) Fluorescence channel (695/55 = SiR) after activation with UV-light to visualize PA-SiR-Halo. (**c**) Coomassie stained gel. Lanes: 1 = ladder (PageRuler Prestained NIR Protein Ladder, ThermoFisher) characteristic masses are given in kDa. 2 = HaloTag, 3 = HaloTag + 0.25 equiv. PA-SiR-Halo, 4 = HaloTag + 0.5 equiv. PA-SiR-Halo, 5 = HaloTag + 0.75 equiv. PA-SiR-Halo, 6 = HaloTag + 1.0 equiv. PA-SiR-Halo, 7 = HaloTag + 1.5 equiv. PA-SiR-Halo. Source data are provided as a Source Data file.

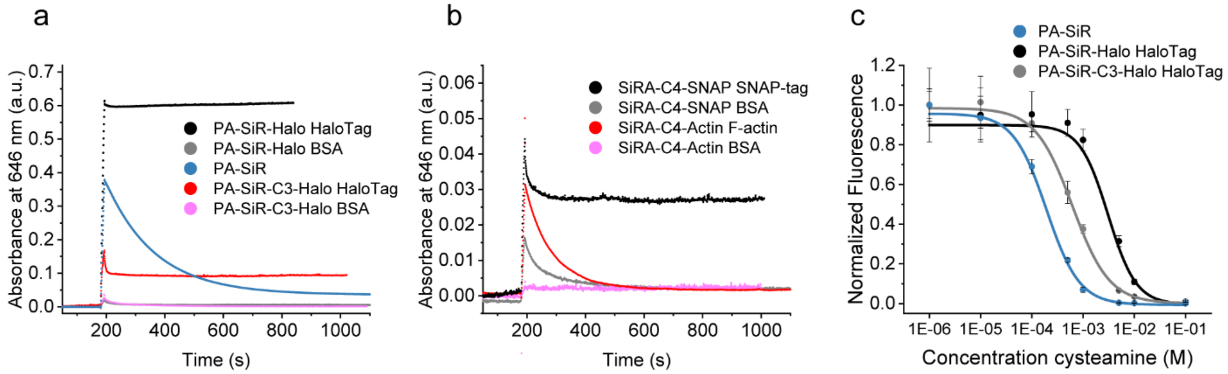

Supplementary Figure 10. Absorbance measurements over time for different PA-SiR probes. Measurements were carried out at 10 µM in PBS at 646 nm. Activation was performed for 12 s after 3 min. (**a**) PA-SiR-Halo analogues were measured in the presence of BSA or HaloTag (20 µM). Reaction with HaloTag influences not only the photoactivation but also the kinetics and thermodynamics of the second equilibrium. PA-SiR-C3-Halo bearing one methylene group less than PA-SiR-Halo showed a fast initial decay after photoactivation followed by a stable signal. In addition, the shortening of the linker was accompanied by a decrease in extinction coefficient (Supplementary Table 1, 5). (**b**) PA-SiR-SNAP and PA-SiR-Actin were measured with the addition of BSA, SNAP-tag (20 µM) or F-actin, respectively. In comparison to the HaloTag probes, PA-SiR-SNAP showed less distinct differences in activation and stability between BSA and SNAP-tag addition conditions. In both cases, nucleophilic attack was observed but the signal of PA-SiR-SNAP conjugated to SNAP-tag decayed to a lesser degree, remaining to a higher degree in the fluorescent form. PA-SiR-Actin did not activate at all in the presence of BSA. Once bound to F-actin it activated better but still decayed over time. This behavior could be due to the probe unbinding from F-actin and subsequently decaying in solution. The structures of the probes investigated in (**a**) and (**b**) are given in Supplementary Fig. 8. (**c**) Fluorescence signal after addition of cysteamine (0.001–100 mM) to fully activated PA-SiR, PA-SiR-Halo and PA-SiR-C3-Halo conjugated to HaloTag (1 µM dye on 2 µM HaloTag) solutions in equilibrium. EC_{50} (half maximal effective concentration) is determined as: 0.192 ± 0.019 mM for PA-SiR (mean ± 95% confidence interval, all N = 24 samples), 3.1 ± 0.5 mM for PA-SiR-Halo conjugated to HaloTag and 0.62 ± 0.06 mM for PA-SiR-C3-Halo conjugated to HaloTag, error bars correspond to 95% confidence intervals. Source data are provided as a Source Data file.

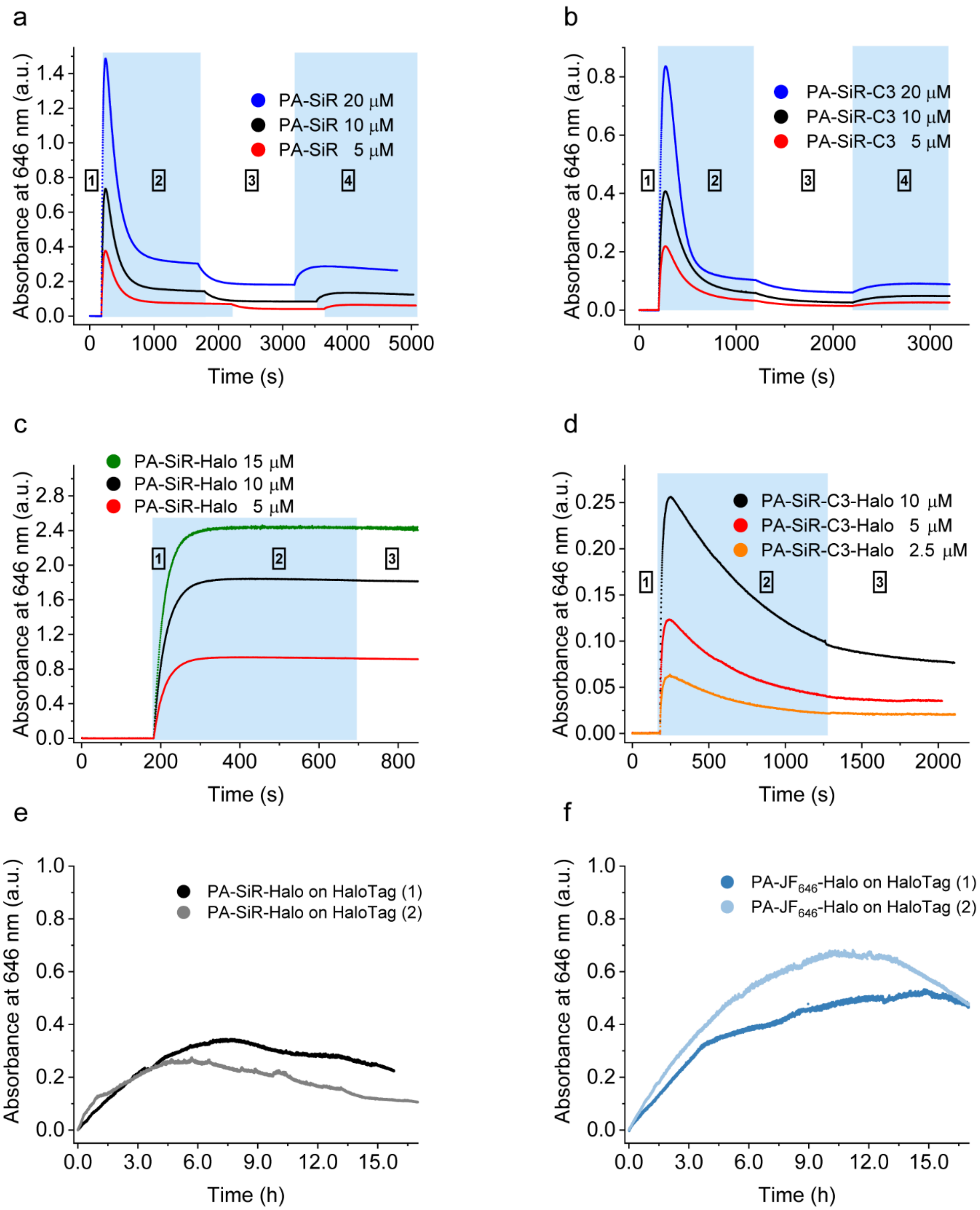

Supplementary Figure 11. Saturation experiment of PA-SiRs at different concentrations. (**a**) PA-SiR. (**b**) PA-SiR-C3. (**c**) PA-SiR-Halo. (**d**) PA-SiR-C3-Halo. Samples were continuously UV irradiated (light blue color) during the first (increase in absorbance) and second section (decrease in absorbance). UV irradiation was discontinued in the third and restarted in the fourth section. Section one was fitted with a mono-

exponential increase to investigate the kinetic parameter k_1 of the photoactivation reaction. Section three was fitted with a mono-exponential decay and the fitted parameters were used to calculate the theoretical extinction coefficient for the photoproducts (Supplementary Table 1, 8-9). (**e**) PA-SiR-Halo (**f**) PA-JF_{646} continuously irradiated with 405 nm light. The data points were fitted with a mono-exponential increase to investigate the kinetic parameter k_1 of the photoactivation reaction (Supplementary Table 1-2, 9).The decrease in absorbance after 8 h, 10 h respectively is attributed to photobleaching and chemical degradation of the fluorophore and labeled protein over time.

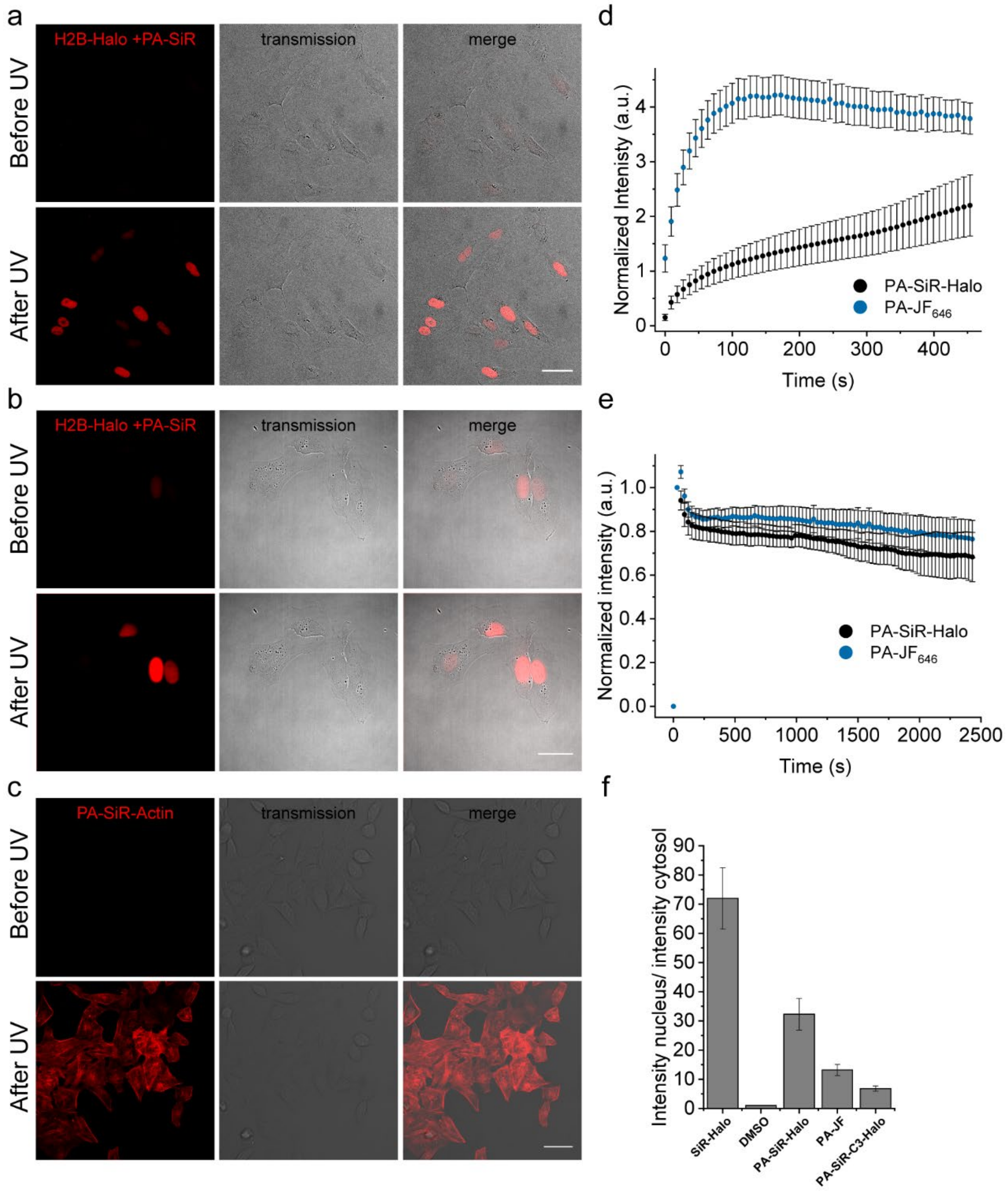

Supplementary Figure 12. Confocal and widefield microscopy images. (**a-b**) Microscopy images of the nucleus in U-2 OS cells transiently expressing H2B-Halo before and after activation with UV light. Stained with PA-SiR-Halo (0.5 µM for 2 h) and (**a**) activated with the DAPI channel (430/35 nm) on a widefield microscope or (**b**) the 355 nm laser on a confocal set up. Scale bars, 40 µm. (**c**) Confocal images of the actin-skeleton in HeLa cells stained with PA-SiR-Actin (1 µM for 1.5 h) before and after UV activation. Scale bar, 40 µm. (**d**) Activation (DAPI channel on widefield) profiles of PA-SiR-Halo and PA-JF_{646} normalized to

the GFP signal in U-2 OS cells transiently expressing H2B-Halo-T2A-EGFP stained with the respective dye (0.5 μM for 2 h). One field of view (FOV) is repeatedly activated with UV light on a widefield set-up (mean ± 95% confidence interval, *N* = 26 cells for PA-SiR-Halo, *N* = 54 cells for PA-JF_{646}-Halo). PA-SiR-Halo shows a 14-fold turn on whereas PA-JF_{646} shows only a 3.5-fold turn on. Source data are provided as a Source Data file. (**e**) Stability of fluorescence signal over time after activation (DAPI channel on widefield) of SiR-Halo and PA-JF_{646}-Halo localized to H2B-Halo-T2A-EGFP (mean ± 95% confidence interval, *N* = 30 cells for PA-SiR-Halo and 70 cells for PA-JF_{646}). Source data are provided as a Source Data file. (**f**) Signal-over-background measurements for different fluorophores (250 nM for 1 h) in U-2 OS cells expressing H2B-Halo-T2A-EGFP (mean ± 95% confidence interval): SiR-Halo (72 ± 11, *N* = 135 cells), DMSO (1.04 ± 0.07, *N* = 126 cells), PA-SiR-Halo (32 ± 5, *N* = 119 cells), PA-JF_{646}-Halo (13.2 ± 1.9, *N* = 121 cells), PA-SiR-C3-Halo (6.8 ± 0.9, *N* = 86 cells), all after activation. Source data are provided as a Source Data file.

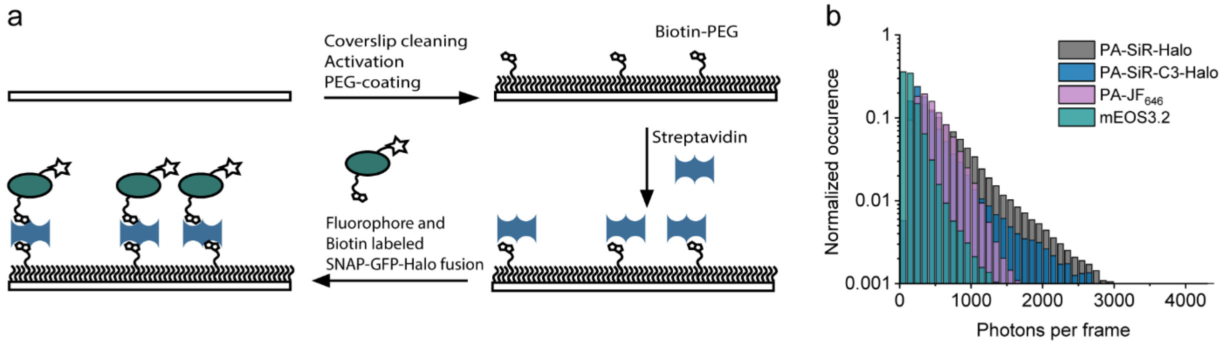

Supplementary Figure 13. Single-molecule assay. (**a**) Schematic view of the *in vitro* TIRF fluorophore assay: A coverslip is cleaned, the surface activated with an amino-silane and then PEG coated. The PEG-brush contains sparse PEG-biotin moieties for fluorophore immobilization. First, PEG-biotin is saturated with streptavidin followed by SNAP:EGFP:Halo or mEOS3.2:Halo labeled with either biotin-BG and fluorophore-chloroalkane or biotin-chloroalkane. (**b**) Photons per particle per frame for PA-SiR-Halo, PA-SiR-C3-Halo and PA-JF_{646}-Halo at 642 nm (1.2 kW cm^{-2}, live-cell tracking regime) and mEOS3.2 at 532 nm (0.9 kW cm^{-2}), respectively. PA-SiR-Halo (668, 518) showed a 30% higher photon output as PA-SiR-C3-Halo (497, 348) and PA-JF_{646}-Halo (474, 414) (mean, median). mEOS3.2 (187, 132), on the other hand, showed much lower photon numbers then the small-molecule fluorophores, as expected from literature.[7] Source data are provided as a Source Data file.

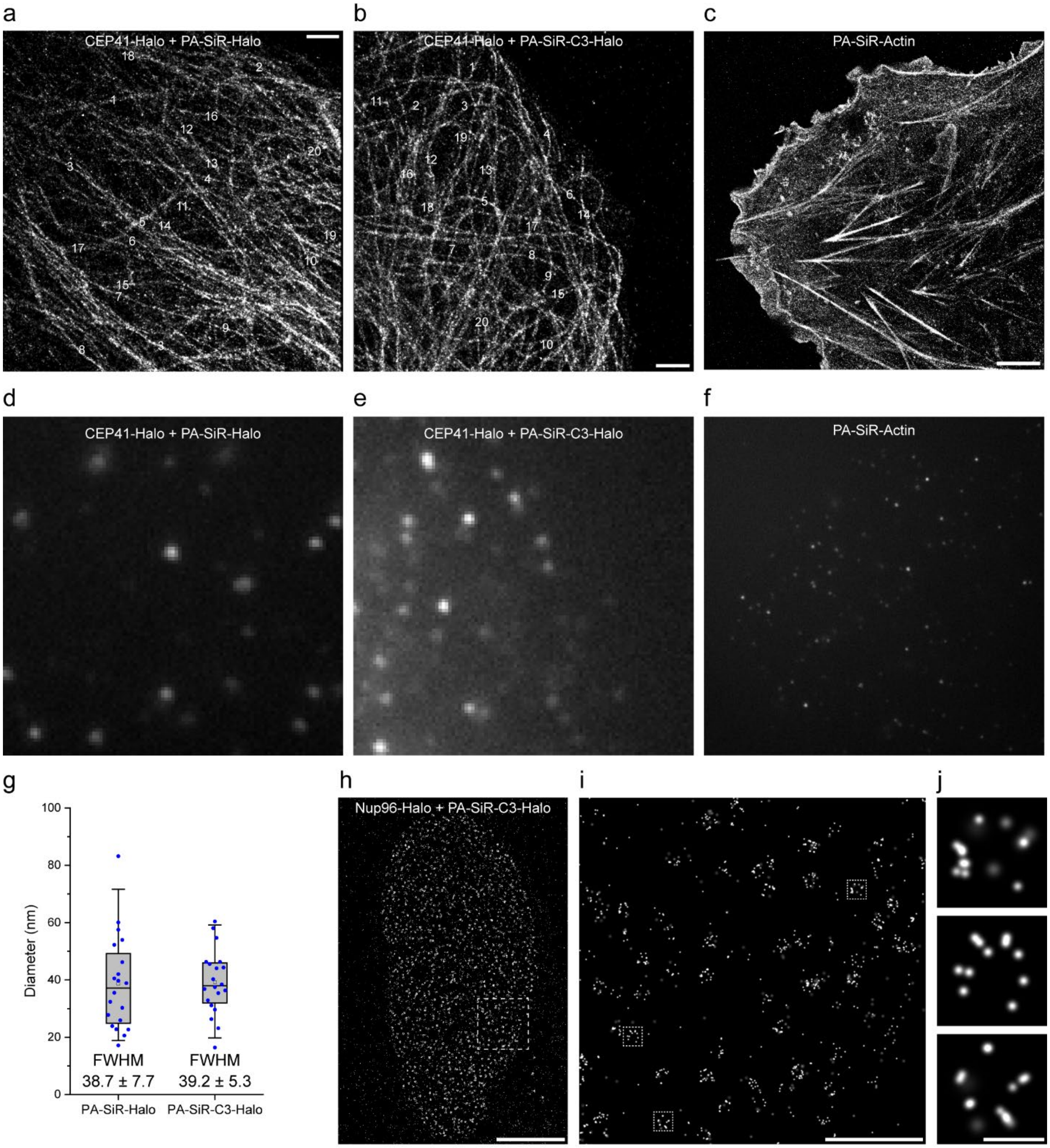

Supplementary Figure 14. Fixed-cell SMLM and quantification. (**a**) Super-resolved image from Figure 3a with the indicated areas for microtubule diameter analysis. Scale bar, 1 µm. (**b**) Super-resolved image of microtubules in a fixed U-2 OS cell, stably expressing CEP41-Halo stained with PA-SiR-C3-Halo (1 µM for 2 h), reconstructed from 6004 frames (100 ms exposure time, 4.2 kW cm^{-2} at 642 nm excitation). Even though the performance of PA-SiR-C3-Halo was not optimal in live-cells (Supplementary Fig. 12f), it was possible to obtain good SMLM images after fixation and washing. The microtubule diameter was found to

be $FWHM_{PA\text{-}SiR\text{-}C3\text{-}Halo}$ = 39.2 ± 5.3 nm (mean ± 95% confidence interval, N = 20 tubules). The areas for microtubule diameter analysis are indicated. Scale bar, 1 µm. (**c**) Super-resolved image of the actin-skeleton in fixed COS-7 cells stained with PA-SiR-Actin (0.5 µM for 2 h). Scale bar, 5 µm. (**d-f**) Single raw camera frames from the recordings to give the super-resolved images above. (**g**) Box-plots of the distribution for the FWHM obtained by fitting the intensity profiles measured along the lines indicated in **a** and **b** (line profiles): box = 25%–75% percentile, whiskers 5%–95% percentile, black line = median, box = mean, individual data points in blue. Source data are provided as a Source Data file. (**h**) Super-resolved overview image of the nuclear pore complex. Endogenously tagged Nup96-Halo in U-2 OS cells was stained with PA-SiR-C3-Halo (1 µM for 2 h). Scale bar, 5 µm. (**i**) Super-resolved image from the boxed region in **h**. Scale bar, 1 µm. (**j**) Single nuclear pores from boxed regions in **i** following the same order. Scale bar, 100 nm.

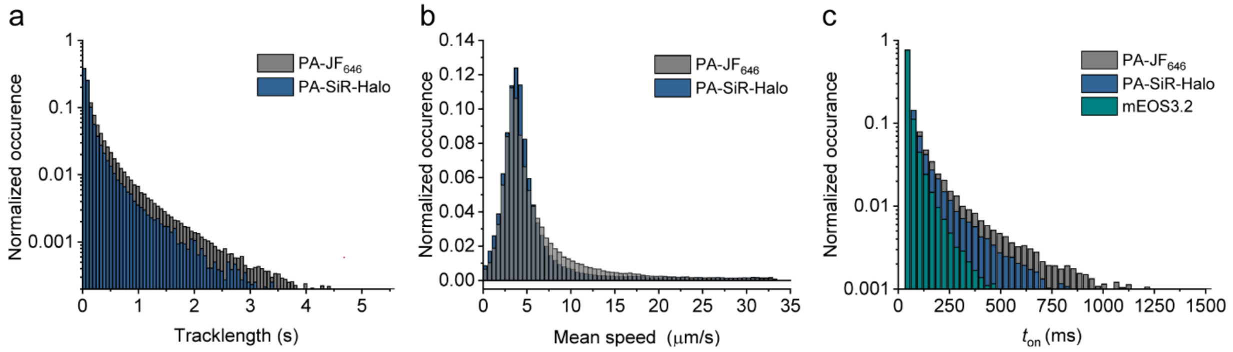

Supplementary Figure 15. Quantification of live-cell tracking experiments in U-2 OS cells. (**a**) Histograms of tracklength found for PA-SiR-Halo (208, statistical mean; no deviation given as not normally distributed; N = 51'408 tracks from 2 FOV) and PA-JF_{646}-Halo (338, N = 233'871 tracks from 2 FOV) labeling β-2-adrenergic-receptor-Halo under identical imaging conditions (30 ms, 0.3 kW cm^{-2} at 642 nm). PA-SiR-Halo displayed similar tracklengths as PA-JF_{646}-Halo and is therefore well suited for live-cell tracking experiments. (**b**) Determined mean speed for PA-SiR-Halo (5.55 ± 0.05 μm s^{-1}, median = 4.1 μm s^{-1}) and PA-JF_{646}-Halo (6.36 ± 0.02 μm s^{-1}, median = 4.4 μm s^{-1}) (mean ± 95% confidence interval, median). (**c**) On-times found in the single-molecule assay for PA-SiR-Halo and PA-JF_{646}-Halo at 642 nm (1.2 kW cm^{-2}, live-cell tracking regime) and mEOS3.2 at 532 nm (0.9 kW cm^{-2}), respectively, showing that the photostability for mEOS3.2 is considerably lower than for the two small-molecule fluorophores. Source data are provided as a Source Data file.

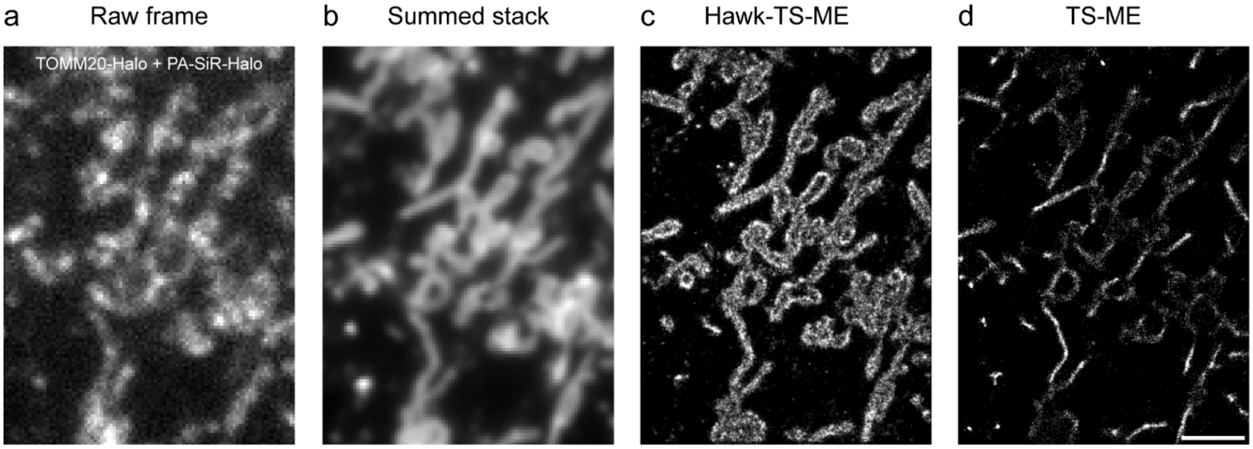

Supplementary Figure 16. Live-cell SMLM experiment. (**a**) Single raw camera frame. (**b**) Sum projection over the first 10 s as depicted in Figure 4b. (**c**) Super-resolved image acquired at high-emitter-density within 10 s (50 ms, 0.3 kW cm^{-2} at 642 nm) from Figure 4a. Processed with the HAWK ImageJ plugin[8] and ThunderSTORM multi-emitter fit[9]. (**d**) Super-resolved image obtained if the data is only processed by ThunderSTORM multi-emitter fit[9], resulting in artificial narrowing and collapsing of structures. Scale bar, 2 µm.

Supplementary Tables

Supplementary Table 1. Spectral properties of different PA-SiR analogues. Results are given as means ± 95% confidence interval. Except for the quantum yield of activation, which is a propagated standard error of the mean. *N* = number of samples measured. Source data are provided as a Source Data file.

	PA-SiR before photoactivation			Photoproduct				
**#**	$\lambda_{abs,max}$ **[nm]**	ε_{max} **[M^{-1}cm^{-1}]**	φ_{act} **%**	$\lambda_{ex,\ max}$/ $\lambda_{em,\ max}$ **[nm]**	$\varepsilon_{max,\ 646\ nm}$ **[M^{-1}cm^{-1}]**	φ **%**	$\tau_{PBS\ or\ BSA}$ **[s]**	$\tau_{on\ POI}$ **[s]**
PA-SiR	276	17’000±<br>3’000<br>*N* = 10	13.3±2.3<br>*N* = 3	646/664	90’000±<br>18’000<br>*N* = 3	19.0±2.4<br>*N* = 3	169±9<br>*N* = 3	N/A
SiR-COOH	N/A	N/A	N/A	645/661<br>*,**	100’000**	39**	N/A	N/A
**20**	314	16’000±<br>5’000<br>*N* = 9	N/D	642/656	N/D	13±10<br>*N* = 3	200±70<br>*N* = 3	N/A
**4**	N/D	N/D	N/D	N/D	N/D	11±8<br>*N* = 3	55±21<br>*N* = 3	N/A
PA-SiR-C3	281	16’000±<br>3’000<br>*N* = 9	29±13<br>*N* = 3	648/668	39’000±<br>7’000<br>*N* = 3	15.6±1.5<br>*N* = 3	271±15<br>*N* = 3	N/A
**33**	N/D	N/D	N/D	N/d	N/D	16.1±0.7<br>*N* = 3	460±60<br>*N* = 3	N/A
PA-SiR-Halo	333	33’000±<br>5’000<br>*N* = 9	0.86±0.07<br>*N* = 3<br>0.09±0.04<br>*N* = 2***	650/666	180’000±<br>30’000<br>*N* = 3	29.2±1.2<br>*N* = 4	40±60<br>*N* = 3	6±3<br>*N* = 3
PA-SiR-C3-Halo	329	20’000±<br>3’000<br>*N* = 9	3.9±0.3<br>*N* = 3	658/672	15’000±<br>3’000<br>*N* = 3	29±5<br>*N* = 3	100±18<br>*N* = 3	7±4<br>*N* = 3
PA-SiR-SNAP	294	19’000±<br>6’000<br>*N* = 10	N/D	650/666	N/D	11.1±2.0<br>*N* = 3	84±19<br>*N* = 3	30±50<br>*N* = 3
PA-SiR-Actin	298	21’000±<br>7’000<br>*N* = 9	N/D	651/666	N/D	3±6<br>*N* = 3	no act	92±3<br>*N* = 3

$\lambda_{abs,max}$ absorption maximum, ε_{max} extinction coefficient, φ_{act} quantum yield of activation, $\lambda_{ex,\ max}$/ $\lambda_{em,\ max}$ excitation and emission maximum, φ quantum yield, τ and $\tau_{on\ POI}$ mean lifetime, N/D not determined, N/A not applicable, * $\lambda_{abs,max}$/ $\lambda_{em,\ max}$, ** from [10], *** at 405 nm.

Supplementary Table 2. Quantum yields of photoactivation for several photoactivatable fluorophores (mean ± standard error of the mean). Source data are provided as a Source Data file.

Name	φ_{act} %	Condition	Citation
PA-SiR	13.3±2.3, *N* = 3	340 nm	This work
PA-SiR-C3	29±13, *N* = 3	340 nm	This work
PA-SiR-Halo on HaloTag	0.86±0.07, *N* = 3	340 nm	This work
PA-SiR-Halo on HaloTag	0.09±0.04, *N* = 2	405 nm	This work
PA-SiR-C3-Halo on HaloTag	3.9±0.3, *N* = 3	340 nm	This work
PA-JF_{549}	2.2	365 nm	[7]
PA-JF_{646}	0.07±0.03, *N* = 2	405 nm	This work
PFF (Fluxional- Rhodamine)	0.4 and 0.5	405 nm pH = 5, 7.4	[11]
mKikGR	0.75	405 nm	[12]
kikGR	0.47	405 nm	[13]

Supplementary Table 3. Fit parameters from Gaussian fit in Fig. 1e.

$$y(x) = y_0 + A /(w \cdot \mathrm{sqrt}(\pi/2)) \cdot e^{-2(x-xc)^2/w^2} \quad (1)$$

Name	y_0 [a.u.]	se_{y0} [a.u.]	xc	se_{xc}	w	se_w	A	se_A	X^2	R^2
A_{eq}	0.000	0.095	**5.339**	0.115	1.972	0.283	2.564	0.560	16.507	0.984

y_0 offset and its standard error se_{y0}; **xc** center and its standard error se_{xc}; w width and its standard error se_w; A area and its standard error se_A; $X^2$the reduced chi-squared and R^2 the squared correlation coefficient are given.

Supplementary Table 4. Fit parameters from sigmoidal fit in Fig. 1e.

$$y(x) = a/(1 + e^{-k(x-xc)}) \quad (2)$$

Name	a [a.u.]	se_a [a.u.]	xc	se_{xc}	k	se_k	X^2	R^2
A_{max}	0.900	0.032	**5.731**	0.084	2.465	0.435	0.004	0.971

a amplitude and its standard error se_a; **xc** center and its standard error se_{xc}; k coefficient and its standard error se_k; $X^2$the reduced chi-squared and R^2 the squared correlation coefficient are given.

Supplementary Table 5. Averaged fit parameters from mono-exponential fits for PA-SiR probes in Fig. 2b and Supplementary Fig. 10a, b. Results are given as means and standard deviations from three measurements. Source data are provided as a Source Data file.

$$y(t) = y_0 + A \cdot e^{(-(t-x)/\tau)} \quad (3)$$

	**Fit Parameters**						**Calculated**						
**Name**	τ [s]	sd_τ [s]	A [a.u.]	sd_A [a.u.]	y_0 [a.u.]	sd_{y0} [a.u.]	A_{eq} [a.u.]	$A_{x=0}$ [a.u.]	K_2	k_{app} [s^{-1}]	k_{-2} [s^{-1}]	k_2 [s^{-1}]	K_{-2}
PA-SiR-Halo BSA	41.98	23.46	0.012	0.001	0.006	0.002	0.006	0.017	1.99	2.38E-02	7.97E-03	1.59E-02	0.50
PA-SiR-Halo Halo	5.74	1.19	0.013	0.004	0.593	0.013	0.593	0.606	0.02	1.74E-01	1.70E-01	3.87E-03	43.99
PA-SiR-C3-Halo BSA	105.02	7.34	0.018	4.4E-04	-0.000*	0.001	0.000	0.018	N/D	9.52E-03	N/D	N/D	N/D
PA-SiR-C3-Halo Halo	6.51	1.55	0.062	0.002	0.095	0.002	0.095	0.157	0.66	1.54E-01	9.27E-02	6.09E-02	1.52
PA-SiR-SNAP BSA	84.10	7.49	0.011	0.001	0.002	0.001	0.002	0.014	4.68	1.19E-02	2.09E-03	9.80E-03	0.21
PA-SiR-SNAP SNAP	32.66	18.20	0.010	0.003	0.026	0.007	0.026	0.036	0.37	3.06E-02	2.23E-02	8.35E-03	2.67
PA-SiR-Actin Actin	92.45	1.35	0.032	0.005	0.002	0.002	0.002	0.035	14.50	1.08E-02	6.98E-04	1.01E-02	0.07

τ decay constant and its standard deviation sd_τ; A amplitude and its standard deviation sd_A; y_0 offset and its standard deviation sd_{y0}, along with the derived parameters: $A_{eq} = y_0$; $A_{x=0} = A + A_{eq}$; $K_2 = (A_{x=0} - A_{eq}) \cdot A_{eq}^{-1}$; $k_{app} = \tau^{-1}$; $k_{-2} = k_{app} \cdot (K_2 + 1)^{-1}$; $k_2 = k_{-2} - k_{app}$; $K_{-1} = k_2 \cdot k_{-2}^{-1}$. *negative equilibrium values are within the experimental error of the instrument and were set to 0 for further calculations. N/D not determined.

Supplementary Table 6. Fit parameters from the dose response fit in Fig. 2c and Supplementary Fig. 10c.

$$y(x) = A1 + (A2-A1)/(1 + 10\text{^}((LOGx0-x) \cdot p)) \quad (4)$$

**Name**	**A1**	se_{A1}	**A2**	se_{A2}	**Logx0**	se_{Logx0}	p	se_p	**EC50**	se_{EC50}	X^2	R^2
PA-SiR	-0.0064	0.0083	0.9568	0.0103	-3.7167	0.0205	-1.3449	0.0647	**1.92E-04**	9.04E-06	0.001	0.997
PA-SiR-Halo	-0.0043	0.0207	0.8989	0.0119	-2.5127	0.0326	-1.5812	0.1251	**3.07E-03**	2.30E-04	0.001	0.991
PA-SiR-C3-Halo	0.0013	0.0108	0.9842	0.0097	-3.2101	0.0186	-1.2019	0.0699	**6.16E-04**	2.64E-05	0.001	0.997

A1 and **A2** asymptotes and their standard errors sd_{A1} and sd_{A2}; **LOGx0** center and its standard error se_{LOGx0}; p hill slope and its standard error se_p; **EC50** derived parameter concentration at half response and its standard error se_{EC50}; X^2 the reduced chi-squared and R^2 the squared correlation coefficient are given.

Supplementary Table 7. Averaged fit parameters from mono-exponential fits in Supplementary Fig. 6 and 7. Results are given as means and standard deviations from three measurements. According to Supplementary Equation (3). Source data are provided as a Source Data file.

	**Fit Parameters**						**Calculated**						
**Name/ pH**	**τ [s]**	**sd_{τ} [s]**	**A [a.u.]**	**sd_A [a.u.]**	**y_0 [a.u.]**	**sd_{y0} [a.u.]**	**A_{eq} [a.u.]**	**$A_{x=0}$ [a.u.]**	**K_2**	**k_{app} [s^{-1}]**	**k_{-2} [s^{-1}]**	**k_2 [s^{-1}]**	**K_{-2}**
PA-SiR PBS	169.50	3.49	0.347	0.008	0.035	0.002	0.035	0.382	9.87	5.90E-03	5.43E-04	5.36E-03	0.10
**20**	202.87	28.89	0.009	0.001	-0.002*	0.001	0.000	0.009	N/D	4.93E-03	N/D	N/D	N/D
**4**	54.92	8.49	0.086	0.002	0.008	0.004	0.008	0.094	10.25	1.82E-02	1.62E-03	1.66E-02	0.10
PA-SiR-C3	271.19	6.02	0.172	0.003	0.013	0.001	0.013	0.185	13.21	3.69E-03	2.59E-04	3.43E-03	0.08
**33**	460.09	22.57	0.057	0.011	0.050	0.007	0.050	0.106	1.14	2.17E-03	1.02E-03	1.16E-03	0.88
10.6	180.19	8.60	0.283	0.014	0.004	0.005	0.004	0.287	74.93	5.55E-03	7.31E-05	5.48E-03	0.01
8.8	171.83	5.29	0.303	0.021	0.010	0.006	0.010	0.313	30.75	5.82E-03	1.83E-04	5.64E-03	0.03
8.1	171.83	4.12	0.310	0.021	0.019	0.006	0.019	0.329	16.63	5.82E-03	3.30E-04	5.49E-03	0.06
7.2	169.55	2.85	0.322	0.045	0.033	0.004	0.033	0.355	9.65	5.90E-03	5.54E-04	5.34E-03	0.10
6.6	114.99	0.65	0.150	0.003	0.121	0.003	0.121	0.271	1.23	8.70E-03	3.90E-03	4.80E-03	0.81
6.2	96.94	2.78	0.094	0.007	0.146	0.001	0.146	0.240	0.64	1.03E-02	6.27E-03	4.04E-03	1.55
5.8	93.18	22.73	0.043	0.004	0.130	0.004	0.130	0.173	0.33	1.07E-02	8.08E-03	2.65E-03	3.05
5.2	97.69	26.62	0.016	0.002	0.057	0.002	0.057	0.072	0.28	1.02E-02	8.02E-03	2.22E-03	3.62
4.8	115.09	65.13	0.009	0.001	0.020	0.002	0.020	0.028	0.45	8.69E-03	6.01E-03	2.68E-03	2.24
4.1	114.50	7.21	0.006	4.2E-04	0.005	2.3E-04	0.005	0.010	1.16	8.73E-03	4.05E-03	4.68E-03	0.87

τ decay constant and its standard deviation **sd_{τ}**; ***A*** amplitude and its standard deviation **sd_A**; ***y_0*** offset and its standard deviation **sd_{y0}**, along with the derived parameters: **$A_{eq} = y_0$; $A_{x=0} = A + A_{eq}$**; **$K_2 = (A_{x=0} - A_{eq}) \cdot A_{eq}^{-1}$**; **$k_{app} = \tau^{-1}$**; **$k_{-2} = k_{app} \cdot (K_2 + 1)^{-1}$**; **$k_2 = k_{-2} - k_{app}$**; **$K_{-1} = k_2 \cdot k_{-2}^{-1}$.** *negative equilibrium values are within the experimental error of the instrument and were set to 0 for further calculations. N/D not determined.

Supplementary Table 8. Fit parameters from mono-exponential fits of the third section for PA-SiR, PA-SiR-C3 (C3) and PA-SiR-C3-Halo (C3Halo) from the saturation experiment in Supplementary Figure 11. Equilibrium values for PA-SiR-Halo (Halo) were taken from the fit parameters from the first section and are displayed here for completeness. The concentration is given in µM. Results are given for one measurement with the standard error of the fit. According to Supplementary Equation (3).

	**Fit Parameters**						**Calculated**								
**Name**	***τ*** **[s]**	**se$_τ$** **[s]**	***A*** **[a.u.]**	**se$_A$** **[a.u.]**	***y$_0$*** **[a.u.]**	**se$_{y0}$** **[a.u.]**	***A*$_{eq}$** **[a.u.]**	***A*$_{x=0}$** **[a.u.]**	***K*$_2$**	***k*$_{app}$** **[s^{-1}]**	***k*$_{-2}$** **[s^{-1}]**	***k*$_2$** **[s^{-1}]**	***K*$_{-2}$**	***K***	***A*$_{sat}$** **[a.u.]**
PA-SiR/5	171.26	0.16	0.028	1.5E-05	0.040	3.9E-06	0.040	0.069	0.69	5.84E-03	3.45E-03	2.39E-03	1.44	9.866	**0.440**
PA-SiR/10	173.14	0.16	0.057	3.3E-05	0.084	6.8E-06	0.084	0.141	0.68	5.78E-03	3.43E-03	2.35E-03	1.46	9.866	**0.911**
PA-SiR/20	172.73	0.12	0.125	4.9E-05	0.182	1.1E-05	0.182	0.307	0.69	5.79E-03	3.42E-03	2.36E-03	1.45	9.866	**1.974**
C3/5	268.05	0.36	0.018	9.0E-06	0.014	6.4E-06	0.014	0.032	1.34	3.73E-03	1.59E-03	2.14E-03	0.74	13.212	**0.192**
C3/10	274.48	0.26	0.031	1.1E-05	0.026	8.1E-06	0.026	0.057	1.23	3.64E-03	1.64E-03	2.01E-03	0.82	13.212	**0.363**
C3/20	269.74	0.27	0.041	1.5E-05	0.059	1.1E-05	0.059	0.101	0.70	3.71E-03	2.18E-03	1.52E-03	1.43	13.212	**0.843**
C3Halo/2.5	388.55	0.79	0.044	4.0E-05	0.020	1.9E-05	0.020	0.064	2.25	2.57E-03	7.93E-04	1.78E-03	0.45	0.657	**0.032**
C3Halo/5	441.00	0.69	0.091	5.6E-05	0.032	3.5E-05	0.032	0.123	2.82	2.27E-03	5.93E-04	1.67E-03	0.35	0.657	**0.053**
C3Halo/10	594.43	1.30	0.196	1.4E-04	0.065	1.3E-04	0.065	0.261	3.02	1.68E-03	4.19E-04	1.26E-03	0.33	0.657	**0.108**
Halo/5	N/A	N/A	N/A	N/A	0.929	1.7E-04	0.929	N/A	N/A	N/A	N/A	N/A	N/A	0.020	**0.947**
Halo/10	N/A	N/A	N/A	N/A	1.832	2.5E-04	1.832	N/A	N/A	N/A	N/A	N/A	N/A	0.020	**1.869**
Halo/15	N/A	N/A	N/A	N/A	2.432	2.5E-04	2.432	N/A	N/A	N/A	N/A	N/A	N/A	0.020	**2.480**

τ decay constant and its standard deviation **sd$_τ$**; ***A*** amplitude and its standard deviation **sd$_A$**; ***y$_0$*** offset and its standard deviation **sd$_{y0}$**, along with the derived parameters: $A_{eq} = y_0$; $A_{x=0} = A + A_{eq}$; $K_2 = (A_{x=0} - A_{eq}) \cdot A_{eq}^{-1}$; $k_{app} = \tau^{-1}$; $k_{-2} = k_{app} \cdot (K_2 + 1)^{-1}$; $k_2 = k_{-2} - k_{app}$; $K_{-1} = k_2 \cdot k_{-2}^{-1}$; ***K*** = equilibrium constant from the Supplementary Table 5 or 7 for the respective compound; $A_{sat} = K \cdot A_{eq} + A_{eq}$. These values where used to calculate the extinction coefficients at 646 nm. N/A not applicable.

Supplementary Table 9. Averaged fit parameters from the exponential fits for the first sections of the saturation experiments. Results are given as means and standard deviations from three/two (405 nm) measurements. According to Supplementary Equation (3). Source data are provided as a Source Data file.

**Name**	**τ [s]**	**sd_τ [s]**	**Wavelength of irradiation**
PA-SiR	19.40	0.36	340 nm
PA-SiR-Halo	28.24	1.99	340 nm
PA-SiR-Halo	12’000	6’000	405 nm
PA-SiR-C3	20.99	0.80	340 nm
PA-SiR-C3-Halo	10.32	0.72	340 nm
PA-JF_{646}	15’000	1’000	405 nm

$\boldsymbol{\tau}$ decay constant and its standard deviation $\mathbf{sd_\tau}$.

Supplementary Table 10. Settings for the different microscopy experiments. In the sixth column exposure time is given for widefield microscopy images and the pixel dwell time for confocal images.

Image	Label	Ligand	Microscope	Excitation [nm]	Exposure time / Pixel dwell time	Activation [nm]	Pinhole	Objective	Size	Fixed-live	Emission [nm]	Comment
Fig. 2d, e	H2B-Halo	PA-SiR-Halo	Confocal	631	0.6 µs	355	1	40x/1.10 water	1024x 1024	Live	777-800	Max projection
Fig. 2f	CEP41-Halo	PA-SiR-Halo	Confocal	631	0.25 µs	355	1	40x/1.10 water	2488x 2488	Live	751-779	
Fig. 2g	LA-Halo	PA-SiR-Halo	Confocal	631	0.475 µs	355	1	40x/1.10 water	1288x 1288	Live	751-779	
Fig. 2h	TOMM20-Halo	PA-SiR-Halo	Confocal	631	1.2 µs	355	1	40x/1.10 water	410x 714	Live	751-779	
Fig. 3a	CEP41-Halo	PA-SiR-Halo	GSD TIRF	642 642/10	100 ms	405 405/10	-	160x/1.43 oil	-	Fixed-MeOH	LP 649 BP 710/100	14’083 frames
Fig. 3b	β-2-adrenergic-receptor-Halo	PA-SiR-Halo	GSD TIRF	642 642/10	30 ms	405 405/10	-	160x/1.43 oil	-	Live	LP 649 BP 710/100	10’046/ 4’000 frames
Fig. 3c-e	NUP96-Halo	PA-SiR-Halo	Widefield (custom)	640 HC Quad	50 ms	405	-	160x/1.43 oil	-	Fixed-FA	700/100	
Fig. 4	TOMM20-Halo	PA-SiR-Halo	GSD TIRF	642 642/10	50 ms	405 405/10	-	160x/1.43 oil	-	Live	LP 649 BP 710/100	1’499 frames
S12a = 2d,e	H2B-Halo	PA-SiR-Halo	Confocal	631	0.6 µs	355	1	40x/1.10 water	1024x 1024	Live	777-800	
S12b	H2B-Halo	PA-SiR-Halo	Widefield	635	500 ms	365	-	40x/1.10 water	-	Live	720/100	
S12c	none	PA-SiR-Actin	Confocal	631	0.225 µs	355	1	63x/1.40 oil	2688x 2688	Live	751-779	
S12d	H2B-Halo	Respective dye	Widefield	635	500 ms	365	-	40x/1.10 water	-	Live	720/100	50 ms act
S12e	H2B-Halo	Respective dye	Widefield	635	500 ms	365	-	40x/1.10 water	-	Live	720/100	Stability
S12f	H2B-Halo	Respective dye	Confocal	631	0.6 µs	355	1	40x/1.10 water	1024x 1024	Live	777-800	Max projection
S13b	Halo:EGFP: SNAP	Respective dye	TIRF	642 642/10	30 ms	405 405/10	-	160x/1.43 oil	400x 400	-	LP 649 BP 710/100	10’000-20’000 frames
S13b	mEOS3.2: Halo	-	TIRF	532 532/10	30 ms	405 405/10	-	160x/1.43 oil	400x 400	-	LP 541 BP 600/100	10’000-20’000 frames
S14a, d = Fig. 3a	CEP41-Halo	PA-SiR-Halo	GSD TIRF	642 642/10	100 ms	405 405/10	-	160x/1.43 oil	-	Fixed-MeOH	LP 649 BP 710/100	14’083 frames
S14b, e	CEP41-Halo	PA-SiR-C3-Halo	TIRF	642 642/10	100 ms	405 405/10	-	160x/1.43 oil	-	Fixed-MeOH	LP 649 BP 710/100	6’004 frames
S14c, f	-	PA-SiR-Actin	GSD TIRF	642 642/10	130 ms	405 405/10	-	160x/1.43 oil	-	Fixed-Glutaraldehyde	LP 649 BP 710/100	30’000 frames
S14h-j	NUP96-Halo	PA-SiR-C3-Halo	Widefield (custom)	640 HC Quad	50 ms	405	-	160x/1.43 oil	-	Fixed-FA	700/100	
S15a,b	β-2-adrenergic-receptor-Halo	Respective dye	GSD TIRF	642 642/10	30 ms	405 405/10	-	160x/1.43 oil	-	Live	LP 649 BP 710/100	

S15c	Halo:EGFP: SNAP	Respective dye	TIRF	642 642/10	30 ms	405 405/10	-	160x/1.43 oil	400x 400	-	LP 649 BP 710/100	10'000-20'000 frames
S15c	mEOS3.2: Halo	-	TIRF	532 532/10	30 ms	405 405/10	-	160x/1.43 oil	400x 400	-	LP 541 BP 600/100	10'000-20'000 frames
S16a-d	TOMM20-Halo	PA-SiR-Halo	GSD TIRF	642 642/10	50 ms	405 405/10	-	160x/1.43 oil	-	Live	LP 649 BP 710/100	1'499 frames

Supplementary Table 11. Details of the crystal structure determination of compound **4**.

	Compound **4**
formula	$C_{20}H_{26}N_2Si$
crystal system	orthorhombic
space group	$P\ 2_12_12_1$
a /Å	9.55658(9)
b /Å	10.42713(10)
c /Å	18.26555(18)
V /Å^3	1820.12(3)
Z	4
M_r	322.52
F_{000}	696
d_c /Mg m^{-3}	1.177
μ /mm^{-1}	0.131
max., min. transmission factors	1.000, 0.483
X-radiation, λ /Å	Mo-K_α, 0.71073
data collect. temperat. /K	120(1)
Θ range /°	2.2 to 34.7
index ranges h,k,l	-15 ... 15, -16 ... 16, -29 ... 29
reflections measured	157476
unique [R_{int}]	7733 [0.0556]
observed [$I \geq 2\sigma(I)$]	7006
data / restraints /parameters	7733 / 0 / 246
GooF on F^2	1.086
R indices [$F>4\sigma(F)$] $R(F)$, $wR(F^2)$	0.0379, 0.0932
R indices (all data) $R(F)$, $wR(F^2)$	0.0452, 0.0961
absolute structure parameter	-0.03(2)
largest residual peaks /e Å^{-3}	0.395, -0.152

Supplementary Methods

Materials and general information for Chemical Synthesis

All chemical reagents and anhydrous solvents for synthesis were purchased from commercial suppliers (Acros, Apollo, Armar, Bachchem, Biomatrik, Fluka, Fluorochem, LC Laboratories, Merck, Reseachem, Roth, Sigma-Aldrich, TCI and TOCRIS) and used without further purification. Halo-NHBoc **24** and BG-NH_2 **27** were synthesized according to literature procedures[14,15]. Jasplakinolide-NHBoc **25** was obtained from a custom synthesis by Spirochrome AG. Composition of mixed solvents is given by volume ratio (v/v). Reactions in the absence of air and moisture were performed in oven-dried glassware under Ar or N_2 atmosphere. Flash column chromatography was performed using a CombiFlash Rf system (Teledyne ISCO) using SiO_2 RediSep® Rf columns at 25 °C or a Biotage (Isolera™) flash system using SiliaSep™ columns. The used solvent compositions are reported individually in parentheses. Analytical thin layer chromatography was performed on glass plates coated with silica gel 60 F254 (Merck). Visualization was achieved using UV light (254 nm). Evaporation *in vacuo* was performed at 25–60 °C and 900–10 mbar. ^1H, ^{13}C, and ^{19}F NMR spectra were recorded on AV 400, Ascend™ 400 and AV 600 Bruker spectrometers at 400 MHz or 600 MHz (^1H), 101 MHz or 151 MHz (^{13}C), 377 MHz or 566 MHz (^{19}F) respectively. All spectra were recorded at 298 K. Chemical shifts δ are reported in ppm downfield from tetramethylsilane using the residual deuterated solvent signals as an internal reference ($CDCl_3$: δ_H = 7.26 ppm, δ_C = 77.16 ppm; CD_3OD: δ_H = 3.31 ppm, δ_C = 49.00 ppm; DMSO-d_6: δ_H = 2.50 ppm, δ_C = 39.52 ppm; CD_3CN: δ_H = 1.94 ppm, δ_C = 118.26 ppm). For ^1H, ^{13}C and ^{19}F NMR, coupling constants *J* are given in Hz and the resonance multiplicity is described as s (singlet), d (doublet), t (triplet), q (quartet), quint (quintet), sext (sextet), sept (septet), m (multiplet) and br. (broad). High-resolution mass spectrometry (HRMS) was performed by the MS-service of the EPF Lausanne (SSMI) on a Waters Xevo® G2-S Q-Tof spectrometer with electron spray ionization (ESI) or by the MS-facility of the Max Planck Institute for Medical Research on a Bruker maXis II™ ETD. Liquid chromatography coupled to mass spectrometry (LC-MS) was performed on a Shimadzu MS2020 connected to a Nexera UHPLC system equipped with a Waters ACQUITY UPLC BEH C18 (1.7 µm, 2.1 x 50 mm) column or a Supelco Titan C18 80 Å (1.9 µm, 2.1 x 50 mm). Buffer A: 0.05% HCOOH in H_2O Buffer B: 0.05%

HCOOH in ACN. Analytical gradient was from 10% to 90% B within 6 min with 0.5 mL min^{-1} flow unless otherwise stated. Preparative reverse phase high-performance liquid chromatography (RP-HPLC) was carried out on a Dionex system equipped with an UltiMate 3000 diode array detector for product visualization on a Waters Symmetry C18 column (5 µm, 3.9 x 150 mm), Waters SunFire™ Prep C18 OBD™ (5 µm, 10 x 150 mm) column, Supleco Ascentis® C18 column (5 µm, 10 x 250 mm) or on a Supleco Ascentis® C18 column (5 µm, 21.2 x 250 mm). Buffer A: 0.1% TFA in H_2O Buffer B: ACN. Typical gradient was from 10% to 90% B within 32 min with 2, 4 or 8 mL min^{-1} flow.

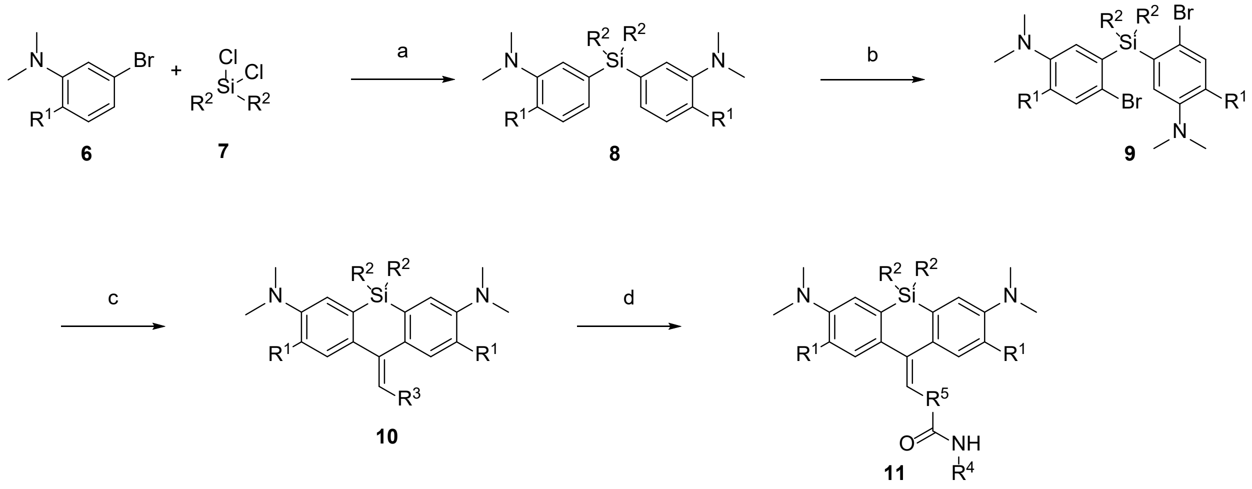

Supplementary Figure 17. Synthetic route to PA-SiR derivatives[16,17]. (a) *sec*-BuLi, Et_2O, –78 °C to room temperature 1 h; (b) NBS, NH_4OAc, ACN, 0 °C to room temperature, 2 h; (c) *sec*-BuLi, THF, anhydride or ester, –78 °C to room temperature 1 h; (d) TSTU, DIPEA, R^4-NH_2, DMSO, room temperature 30 min. R^1 = F or H; R^2 = Me or ^{i}Pr; R^3 = H, CH_2CH_3, CH_2COOH or CH_2CH_2COOH; R^4 = BG, chloroalkane, jasplakinolide; R^5 = CH_2CH_2 or CH_2.

General procedure A for the silane introduction

3-Bromo-*N,N*-dimethylaniline (**12**) (3.20 g, 16.00 mmol, 2.0 eq.) was dissolved in dry Et_2O (45 mL) and cooled down to –78 °C. *sec*-BuLi (14.0 mL, 18.40 mmol, 2.3 eq., 1.3 M in cyclohexane) was added dropwise over 15 min and the mixture was stirred for 30 min at –78 °C. Dichlorodimethylsilane (**13**) (1.0 mL, 8.00 mmol, 1.0 eq.) was added dropwise over 10 min at –78 °C. The mixture was stirred for 10 min at –78 °C and then warmed up to room temperature and stirred for 1 h. The mixture was quenched with aqueous saturated $NaHCO_3$ solution. The

aqueous layer was extracted with Et_2O (3 x 150 mL) and the combined organic layers were dried over $MgSO_4$, filtered and evaporated to afford the crude product.

General procedure B for the bromination

A solution of **14** (1.85 g, 6.18 mmol, 1.0 eq.) and ammonium acetate (95 mg, 1.24 mmol, 0.2 eq.) in ACN (30 mL) was cooled down to 0 °C. NBS (2.3 g, 12.98 mmol, 2.1 eq.) was added portion wise over 10 min. The mixture was stirred at 0 °C for 30 min and then warmed up to room temperature and stirred for 2 h. A mixture of aqueous saturated $NaHCO_3$ solution and water 1:1 was added. The aqueous layer was extracted with CH_2Cl_2 (3 x 100 mL) and the combined organic layers were dried over $MgSO_4$, filtered and evaporated to afford the crude product.

General procedure C for the ring closure

A solution of **15** (365 mg, 0.8 mmol, 1.0 eq.) in dry THF (8 mL) was cooled down to –78 °C. *sec*-BuLi (1.4 mL, 1.76 mmol, 2.2 eq., 1.3 M in cyclohexane) was added dropwise over 5 min and the mixture was stirred for 30 min at –78 °C. A solution of glutaric anhydride (**16**) (100 mg, 0.88 mmol, 1.1 eq.) in dry THF (1.0 mL) was added to the mixture. The mixture was stirred at –78 °C for 15 min and then warmed up to room temperature and stirred for 30 min. Acetic acid (2 mL) was added to the mixture. The blue mixture was adsorbed on SiO_2 (2 g).

3,3'-(Dimethylsilanediyl)bis(*N*,*N*-dimethylaniline) 14

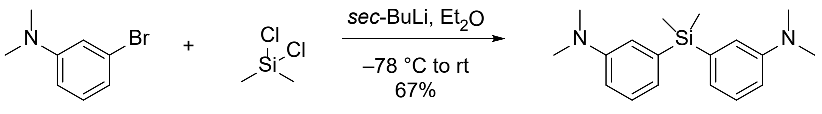

12 13 14

Supplementary Figure 18. Synthetic route. Reaction to give compound **14**.

Following general procedure **A**, flash column chromatography (SiO_2, hexane/EtOAc 100:0 → 70:30) gave **14** (1.598 g, 67%) as a colorless oil.

^1H NMR (400 MHz, $CDCl_3$): δ 7.22 – 7.31 (m, 2H), 6.92 – 6.98 (m, 4H), 6.78 (ddd, J = 8.3, 2.8, 1.0 Hz, 2H), 2.94 (s, 12H), 0.56 (s, 6H); ^{13}C NMR (101 MHz, $CDCl_3$): δ 150.0, 139.1, 128.6, 122.9, 118.5, 113.7, 40.8, –2.0; HRMS (*m/z*): $[M + H]^+$ calcd. for $C_{18}H_{27}N_2Si^+$, 299.1938; found, 299.1940.

3,3'-(Dimethylsilanediyl)bis(4-bromo-*N*,*N*-dimethylaniline) 15

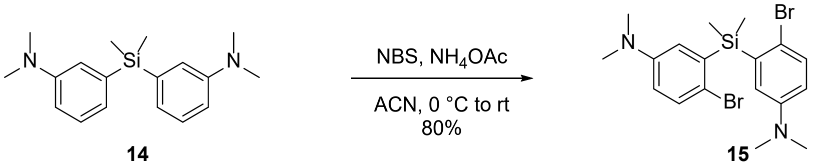

Supplementary Figure 19. Synthetic route. Reaction to give compound **15**.

Following general procedure **B**, flash column chromatography (SiO_2, hexane/CH_2Cl_2 100:0 → 0:100) gave **15** (2.270 g, 80%) as a beige solid.

^{1}H NMR (400 MHz, $CDCl_3$): δ 7.35 (d, J = 8.7 Hz, 2H), 6.84 (d, J = 3.2 Hz, 2H), 6.60 (dd, J = 8.7, 3.2 Hz, 2H), 2.88 (s, 12H), 0.75 (s, 6H); ^{13}C NMR (101 MHz, $CDCl_3$): δ 149.0, 138.9, 133.1, 121.9, 116.9, 115.4, 40.7, –0.8; HRMS (m/z): $[M + H]^+$ calcd. for $C_{18}H_{25}Br_2N_2Si^+$, 455.0148; found, 455.0145.

10-(3-Carboxypropylidene)-7-(dimethylamino)-*N*,*N*,5,5-tetramethyl-5,10-dihydrodibenzo[*b*,*e*]-silin-3-aminium trifluoroacetate PA-SiR (1)

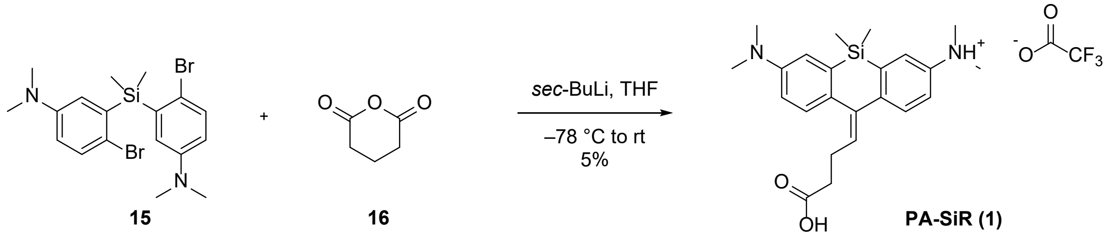

Supplementary Figure 20. Synthetic route. Reaction to give PA-SiR.

Following general procedure **C**, flash column chromatography (SiO_2, CH_2Cl_2/MeOH 100:0 → 90:10) and RP-HPLC (4 mL min^{-1}, 10% to 90% B in 32 min) gave PA-SiR (23 mg, 5%) as a light green solid.

^{1}H NMR (400 MHz, CD_3OD): δ 7.70 (t, J = 2.9 Hz, 2H), 7.66 (d, J = 8.5 Hz, 1H), 7.61 (d, J = 8.4 Hz, 1H), 7.55 (dd, J = 8.5, 2.6 Hz, 1H), 7.50 (dd, J = 8.5, 2.7 Hz, 1H), 6.03 (t, J = 7.3 Hz, 1H), 3.27 (s, 6H), 3.26 (s, 6H), 2.68 (q, J = 7.4 Hz, 2H), 2.45 (t, J = 7.2 Hz, 2H), 0.52 (br. s, 6H); ^{13}C NMR (101 MHz, CD_3OD): δ 176.3, 162.3 (q, J = 36.0 Hz), 150.1, 144.5, 143.4, 143.2, 141.0, 140.3, 138.5, 134.1, 131.3, 128.6, 123.8, 123.7, 121.7, 120.0, 117.8 (q, J = 291.0 Hz), 46.4, 45.7, 34.8, 26.6, –3.8;

^{19}F NMR (376 MHz, CD_3OD): δ –77.11; HRMS (*m/z*): [M – H]$^-$ calcd. for $C_{23}H_{29}N_2O_2Si^-$, 393.2004; found, 393.1992.

Note: All attempts (HPLC: Triethylammonium acetate/ACN pH = 8; triethylammonium bicarbonate/ACN pH = 8 buffer system) to isolate the fluorescent SiR were not successful.

3-((2-(4-Carboxybutanoyl)-5-(dimethylamino)phenyl)dimethylsilyl)-*N*,*N*-dimethylbenzen-aminium trifluoroacetate 17

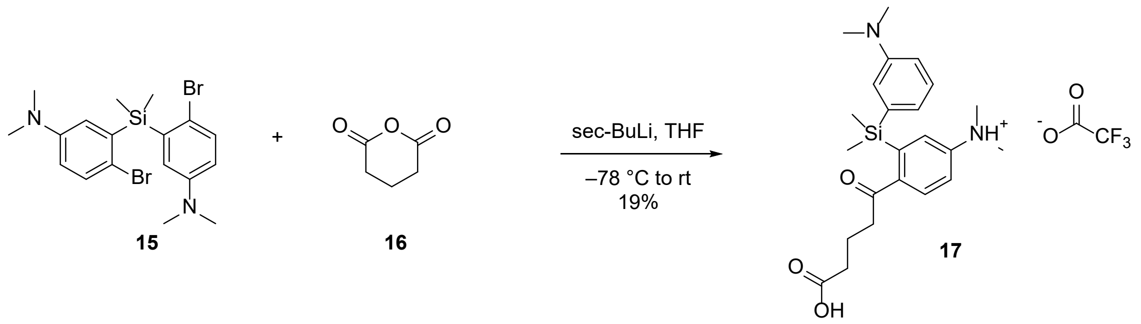

Supplementary Figure 21. Synthetic route. Major side product, compound **17**, formed in the reaction to give PA-SiR.

Compound **17** is the major side product isolated during preparation of PA-SiR (**1**). Following general procedure **C**, flash column chromatography (SiO_2, hexane/EtOAc 100:0 → 0:100 then CH_2Cl_2/MeOH 100:0 → 90:10) and RP-HPLC (8 mL min^{-1}, 10% to 90% B in 32 min) also gives **17** (65 mg, 19%) as a light blue solid.

^1H NMR (400 MHz, CD_3OD): δ 7.97 (d, J = 8.9 Hz, 1H), 7.67 (dd, J = 2.6, 0.8 Hz, 1H), 7.51 – 7.58 (m, 2H), 7.48 (dd, J = 8.2, 7.0 Hz, 1H), 7.02 (d, J = 2.8 Hz, 1H), 6.79 (dd, J = 8.9, 2.7 Hz, 1H), 3.27 (s, 6H), 3.05 (s, 6H), 2.86 (t, J = 7.2 Hz, 2H), 2.12 (t, J = 7.4 Hz, 2H), 1.76 (p, J = 7.3 Hz, 2H), 0.57 (s, 6H); ^{13}C NMR (101 MHz, CD_3OD): δ 200.2, 176.9, 161.92 (d, J = 36.1 Hz), 153.9, 147.4, 143.4, 141.6, 136.2, 133.7, 130.6, 130.3, 126.0, 121.6, 120.4, 117.63 (q, J = 290.4 Hz), 112.3, 47.2, 40.1, 37.3, 33.9, 21.5, -0.3; ^{19}F NMR (376 MHz, CD_3OD): δ –77.15 ppm; HRMS (*m/z*): [M + 2H]$^{2+}$ calcd. for $C_{23}H_{34}N_2O_3Si^{2+}$, 207.1164; found, 207.1163.

N^3,N^3,N^7,N^7,5,5-Hexamethyl-10-methylene-5,10-dihydrodibenzo[*b*,*e*]siline-3,7-diamine 4

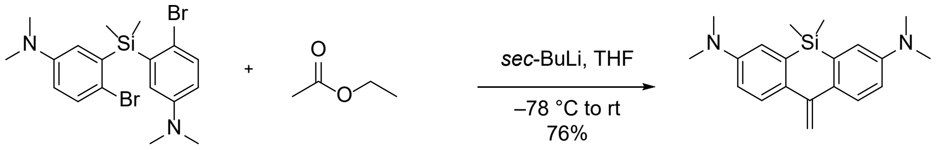

Supplementary Figure 22. Synthetic route. Reaction to give compound **4**.

Following general procedure **C**, washing the solid with MeOH gave **4** (195 mg, 76%) as a white solid.

^{1}H NMR (400 MHz, $CDCl_3$): δ 7.59 (d, J = 8.7 Hz, 2H), 6.93 (d, J = 2.8 Hz, 2H), 6.79 (dd, J = 8.7, 2.8 Hz, 2H), 5.41 (s, 2H), 2.99 (s, 12H), 0.44 (s, 6H); ^{13}C NMR (101 MHz, $CDCl_3$): δ 149.2, 147.7, 135.2, 134.8, 126.9, 115.9, 114.2, 111.3, 40.9, –2.1; HRMS (*m/z*): $[M + H]^+$ calcd. for $C_{20}H_{27}N_2Si^+$, 323.1938; found, 323.1936.

7-(Dimethylamino)-*N*,*N*,5,5-tetramethyl-10-propylidene-5,10-dihydrodibenzo[*b*,*e*]silin-3-aminium trifluoroacetate 20

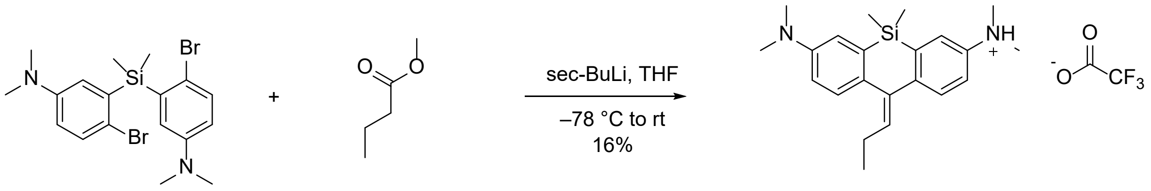

Supplementary Figure 23. Synthetic route. Reaction to give compound **20**.

Following general procedure **C**, flash column chromatography (SiO_2, hexane/EtOAc 90:10 → 70:30) and RP-HPLC (8 mL min^{-1}, 20% to 100% B in 32 min) gave **20** (61 mg, 16%) as a light blue solid.

^{1}H NMR (400 MHz, CD_3OD): δ 7.60 – 7.68 (m, 2H), 7.60 (d, J = 2.7 Hz, 1H), 7.47 – 7.54 (m, 2H), 7.40 (dd, J = 8.5, 2.7 Hz, 1H), 6.01 (t, J = 7.5 Hz, 1H), 3.25 (s, 6H), 3.22 (s, 6H), 2.39 (p, J = 7.5 Hz, 2H), 1.07 (t, J = 7.5 Hz, 1H), 0.50 (br. s, 6H); ^{13}C NMR (101 MHz, CD_3OD): δ 162.2 (q, J = 36.0 Hz), 150.0, 145.0, 143.6, 142.4, 139.8, 139.7, 138.3, 137.2, 131.2, 128.4, 123.3, 122.8, 121.3, 119.2,

117.7 (q, J = 283.5 Hz), 46.1, 45.2, 24.4, 14.6, –3.8; ^{19}F NMR (376 MHz, CD_3OD): δ –77.15; HRMS (*m/z*): $[M + 2H]^{2+}$ calcd. for $C_{22}H_{32}N_2Si^{2+}$, 176.1162; found, 176.1160.

3,7-Bis(dimethylamino)-5,5-dimethyl-3',4'-dihydro-5*H*,5'*H*-spiro[dibenzo[*b*,*e*]siline-10,2'-furan]-5'-one 22

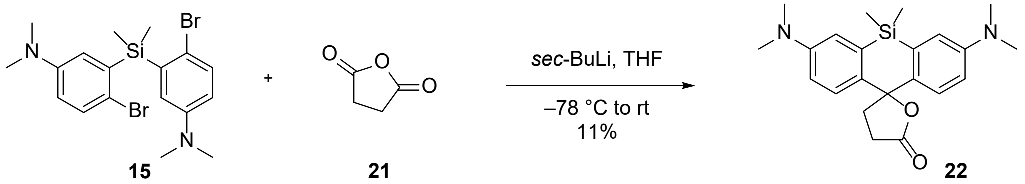

Supplementary Figure 24. Synthetic route. Reaction to give compound **22**.

A solution of **15** (296 mg, 0.65 mmol, 1.0 eq.) in dry THF (9 mL) was cooled down to –78 °C. *sec*-BuLi (1.2 mL, 1.7 mmol, 2.6 eq., 1.3 M in cyclohexane) was added dropwise over 5 min and the mixture was stirred for 30 min at –78 °C. A solution of succinic anhydride (**21**) (72 mg, 0.72 mmol, 1.1 eq.) in dry THF (2.0 mL) was added to the mixture. The mixture was stirred at –78 °C for 15 min and then warmed up to room temperature and stirred for 30 min. Aqueous saturated NH_4Cl was added and the aqueous layer was extracted with EtOAc (2 x 25 mL). The combined organic layers were dried over $MgSO_4$, filtered and evaporated to afford the crude product. Flash column chromatography (SiO_2, hexane/EtOAc 100:0 → 0:100) gave **22** (28 mg, 11%) as a white solid.

^{1}H NMR (400 MHz, CD_3CN): δ 7.34 (d, J = 8.8 Hz, 2H), 7.06 (d, J = 2.8 Hz, 2H), 6.79 (dd, J = 8.8, 2.9 Hz, 2H), 2.95 (s, 12H), 2.52 (td, J = 7.9, 0.7 Hz, 2H), 2.30 (td, J = 8.4, 0.7 Hz, 2H), 0.58 (s, 3H), 0.42 (s, 3H); ^{13}C NMR (101 MHz, CD_3CN): δ 178.6, 150.2, 138.8, 134.8, 124.2, 118.6, 114.3, 88.7, 42.9, 40.7, 29.2, 0.5, –2.4; HRMS (m/z): $[M + H]^+$ calcd. for $C_{22}H_{29}N_2O_2Si^+$, 381.1993; found, 381.1993.

10-(2-Carboxyethylidene)-7-(dimethylamino)-*N*,*N*,5,5-tetramethyl-5,10-dihydrodibenzo[b,e]-silin-3-aminium trifluoroacetate PA-SiR-C3 (23)

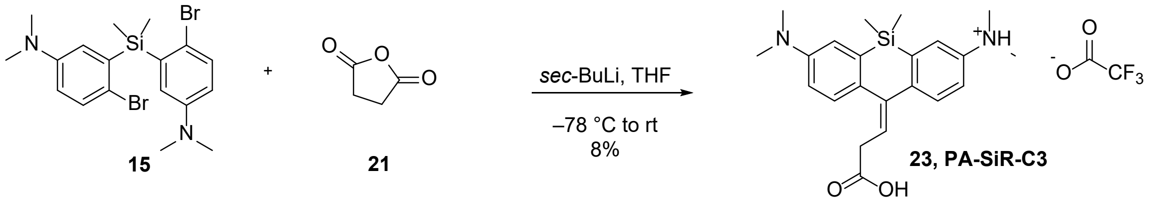

Supplementary Figure 25. Synthetic route. Reaction to give PA-SiR-C3.

A solution of **15** (296 mg, 0.65 mmol, 1.0 eq.) in dry THF (9 mL) was cooled down to –78 °C. *sec*-BuLi (1.2 mL, 1.7 mmol, 2.6 eq., 1.3 M in cyclohexane) was added dropwise over 5 min and the mixture was stirred for 30 min at –78 °C. A solution of succinic anhydride (**21**) (72 mg, 0.72 mmol, 1.1 eq.) in dry THF (2.0 mL) was added to the mixture. The mixture was stirred at –78 °C for 15 min and then warmed up to room temperature and stirred for 30 min. Aqueous saturated NH_4Cl was added and the aqueous layer was extracted with EtOAc (2 x 25 mL). The combined organic layers were dried over $MgSO_4$, filtered and evaporated to afford the crude product. Flash column chromatography (SiO_2, hexane/EtOAc 100:0 → 0:100) gave **22** (28 mg, 11%) as a white solid. The compound was dissolved in water with 0.1% TFA. The blue solution was frozen and lyophilized overnight. RP-HPLC (8 mL min^{-1}, 20% to 90% B in 32 min) gave PA-SiR-C3 (26.5 mg, 8%) as a light blue solid.

^{1}H NMR (400 MHz, CD_3CN): δ 7.57 – 7.60 (m, 2H), 7.48 (d, J = 2.7 Hz, 1H), 7.43 (d, J = 8.5 Hz, 1H), 7.40 (dd, J = 8.5, 2.6 Hz, 1H), 7.23 (dd, J = 8.5, 2.7 Hz, 1H), 6.06 (t, J=7.6 Hz, 1H), 3.33 (d, J = 7.7 Hz, 2H), 3.11 (s, 6H), 3.08 (s, 6H), 0.45 (s, 6H); ^{13}C NMR (101 MHz, CD_3CN): δ 173.1, 147.4, 146.2, 144.4, 142.3, 139.0, 138.6, 137.5, 130.4, 127.8, 125.1, 123.1, 121.9, 120.9, 118.5, 45.3, 43.9, 35.6, –3.7; ^{19}F NMR (376 MHz, CD_3CN): δ –76.26; HRMS (*m/z*): $[M + 2H]^{2+}$ calcd. for $C_{22}H_{30}N_2O_2Si^{2+}$, 191.1033; found, 191.1032.

Note: For reaction with succinic anhydride both PA-SiR-C3 and compound **22** could be isolated. Analogues work up and isolation procedure for PA-SiR did not lead to the isolation of the spirolactone but only PA-SiR.

10-(4-((2-(2-((6-Chlorohexyl)oxy)ethoxy)ethyl)amino)-4-oxobutylidene)-7-(dimethylamino)-*N*,*N*,5,5-tetramethyl-5,10-dihydrodibenzo[*b*,*e*]silin-3-aminium trifluoroacetate PA-SiR-Halo (5)

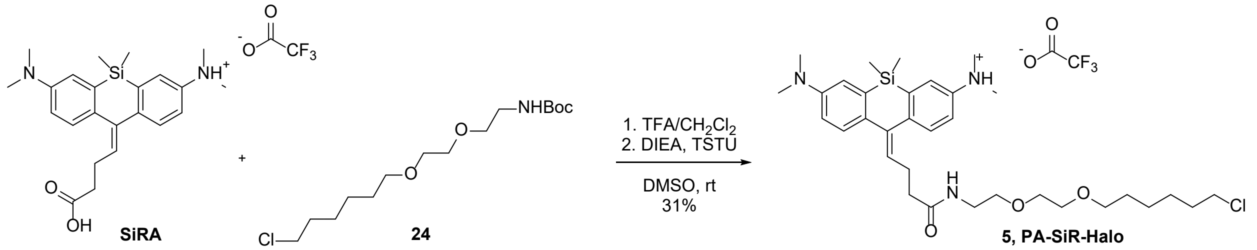

Supplementary Figure 26. Synthetic route. Reaction to give PA-SiR-Halo.

A solution of PA-SiR (4.0 mg, 8.0 µmol, 1.0 eq.) in DMSO (300 µL) was treated with DIEA (5.0 µL, 30.1 µmol, 3.8 eq.) and TSTU (3.6 mg, 12.0 µmol, 1.5 eq.). The mixture was shaken for 20 min at room temperature. In a separate vial a solution of Halo-NHBoc **24** (4.0 mg, 12.3 µmol, 1.5 eq.) in TFA/CH_2Cl_2 (2:8, 80 µL) was shaken for 5 min. The solution was evaporated and dried on the high vacuum for 1 h. The residue was taken up in DMSO (50 µL) and added to the other mixture. The mixture was shaken for 10 min and then acidified with TFA (3 µL). RP-HPLC (4 mL min^{-1}, 10% to 90% B in 32 min) gave PA-SiR-Halo (1.8 mg, 31%) as a light blue solid.

^{1}H NMR (400 MHz, CD_3OD): δ 7.61 (d, J = 8.5 Hz, 1H), 7.48 – 7.56 (m, 3H), 7.41 (dd, J = 8.5, 2.7 Hz, 1H), 7.30 (dd, J = 8.5, 2.7 Hz, 1H), 5.95 (t, J = 7.2 Hz, 1H), 3.48 – 3.52 (m, 8H), 3.45 (t, J = 6.5 Hz, 2H), 3.33 (d, J = 5.7 Hz, 2H), 3.22 (s, 6H), 3.19 (s, 6H), 2.69 (q, J = 7.3 Hz, 2H), 2.36 (t, J = 7.3 Hz, 2H), 1.73 (p, J = 6.9 Hz, 2H), 1.56 (p, J = 6.8 Hz, 2H), 1.30 – 1.47 (m, 4H), 0.49 (s, 6H); ^{13}C NMR (101 MHz, CD_3OD): δ 175.0, 163.2 (d, J = 34.2 Hz), 148.7, 145.9, 144.4, 141.3, 139.6, 138.8, 138.2, 132.7, 131.1, 128.4, 122.5, 121.9, 120.6, 118.4, 117.7 (d, J = 291.1 Hz), 72.2, 71.2, 71.2, 70.6, 45.7, 45.6, 44.5, 40.4, 36.9, 33.7, 30.5, 27.7, 27.6, 26.5, –7.1; ^{19}F NMR (376 MHz, CD_3OD): δ –77.21; HRMS (*m/z*): [M + H]$^{+}$ calcd. for $C_{33}H_{51}ClN_3O_3Si^{+}$, 600.3383; found, 600.3386.

10-(4-((4-((4*R*,7*R*,10*S*,13*S*,19*S*,*E*)-7-((1*H*-Indol-2-yl)methyl)-4-(4-hydroxyphenyl)-8,13,15,19-tetramethyl-2,6,9,12-tetraoxo-1-oxa-5,8,11-triazacyclononadec-15-en-10-yl)butyl)amino)-4-oxobutylidene)-7-(dimethylamino)-*N*,*N*,5,5-tetramethyl-5,10-dihydrodibenzo[*b*,*e*]silin-3-aminium trifluoroacetate PA-SiR-Actin (26)

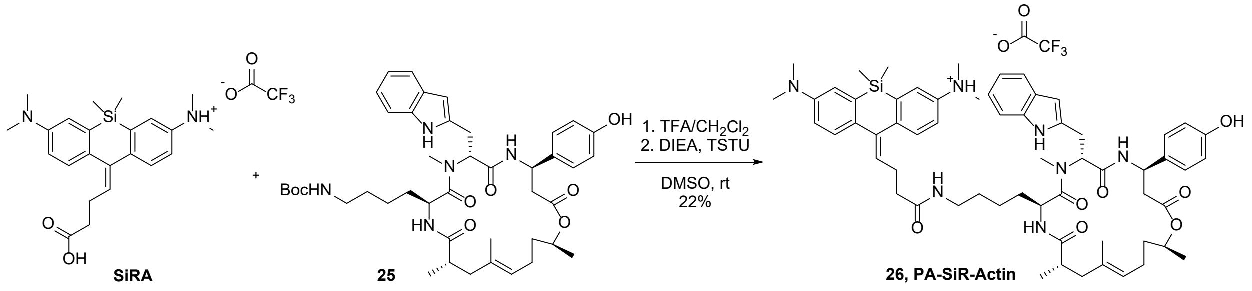

Supplementary Figure 27. Synthetic route. Reaction to give PA-SiR-Actin.

A solution of PA-SiR (4.0 mg, 8.0 µmol, 1.0 eq.) in DMSO (300 µL) was treated with DIEA (5.0 µL, 30.1 µmol, 3.8 eq.) and TSTU (3.6 mg, 12.0 µmol, 1.5 eq.). The mixture was shaken for 20 min at room temperature. In a separate vial a solution of jasplakinolide-NHBoc **25** (9.5 mg, 12.3 µmol, 1.5 eq.) in TFA/CH_2Cl_2 (2:8, 80 µL) was shaken for 2 min. The solution was evaporated and dried on the high vacuum for 1 h. The residue was taken up in DMSO (50 µL) and added to the other mixture. The mixture was shaken for 30 min and then acidified with TFA (3 µL). RP-HPLC (4 mL min^{-1}, 10% to 90% B in 32 min) gave PA-SiR-Actin (2.6 mg, 22%) as a light blue solid.

^{1}H NMR (400 MHz, CD_3OD): δ 8.42 (d, J = 8.6 Hz, 1H), 7.64 (d, J = 8.5 Hz, 1H), 7.53 – 7.61 (m, 4H), 7.47 (dd, J = 8.4, 2.7 Hz, 1H), 7.32 – 7.37 (m, 1H), 7.24 – 7.30 (m, 1H), 6.95 – 7.10 (m, 5H), 6.72 – 6.81 (m, 2H), 5.96 (t, J = 7.3 Hz, 1H), 5.60 (dd, J = 10.1, 6.4 Hz, 1H), 5.27 (dt, J = 9.8, 5.0 Hz, 1H), 5.04 (t, J = 7.0 Hz, 1H), 4.78 – 4.85 (m, 1H), 4.67 (t, J = 5.7 Hz, 1H), 3.23 (s, 6H), 3.18 (s, 6H), 2.99 – 3.15 (m, 4H), 2.93 (s, 3H), 2.66 – 2.79 (m, 4H), 2.61 (ddd, J = 10.2, 6.8, 3.0 Hz, 1H), 2.35 (t, J = 7.2 Hz, 2H), 2.26 – 2.33 (m, 1H), 1.84 – 1.95 (m, 3H), 1.57 – 1.64 (m, 1H), 1.55 (s, 3H), 1.41 (td, J = 13.3, 7.7 Hz, 1H), 1.23 (s, 2H), 1.18 (d, J = 6.3 Hz, 3H), 1.07 (d, J = 6.8 Hz, 3H), 0.95 (d, J = 5.5 Hz, 2H), 0.86 (d, J = 8.1 Hz, 2H), 0.53 (d, J = 3.6 Hz, 6H); ^{13}C NMR (101 MHz, CD_3OD): δ 178.0, 174.9, 174.4, 172.3, 171.7, 157.8, 149.0, 145.8, 144.2, 141.3, 139.6, 138.3, 138.0, 135.4, 134.7, 133.6, 132.9, 131.1, 128.5, 128.4, 128.2, 125.8, 124.4, 122.9, 122.4, 120.9, 119.8, 119.5, 119.0, 118.6, 116.3, 112.3, 110.7, 71.9, 57.4, 50.7, 50.1, 45.7, 44.6, 44.5, 41.9, 40.3, 40.1, 37.0, 36.6, 32.6, 31.3,

30.0, 27.5, 25.9, 24.6, 23.2, 20.3, 20.0, 16.6, –0.2; ^{19}F NMR (376 MHz, CD_3OD): δ –77.26; HRMS (*m/z*): $[M + H]^+$ calcd for $C_{61}H_{80}N_7O_7Si^+$, 1050.5883; found, 1050.5902.

10-(4-((4-(((2-Amino-9*H*-purin-6-yl)oxy)methyl)benzyl)amino)-4-oxobutylidene)-7-(dimethyl-amino)-*N*,*N*,5,5-tetramethyl-5,10-dihydrodibenzo[*b*,*e*]silin-3-aminium trifluoroacetate PA-SiR-SNAP (28)

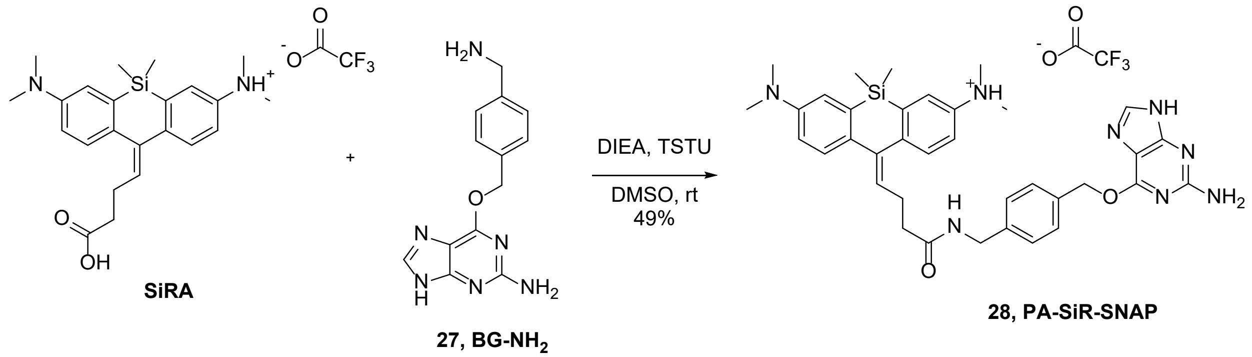

Supplementary Figure 28. Synthetic route. Reaction to give PA-SiR-SNAP.

A solution of PA-SiR (4.0 mg, 8.0 µmol, 1.0 eq.) in DMSO (300 µL) was treated with DIEA (5.0 µL, 30.1 µmol, 3.8 eq.) and TSTU (3.6 mg, 12.0 µmol, 1.5 eq.). The mixture was shaken for 20 min and BG-NH_2 **27** (3.4 mg, 12.8 µmol, 1.6 eq.) was added. The mixture was shaken for 10 min at room temperature and then acidified with TFA (15 µL). RP-HPLC (8 mL min^{-1}, 10% to 90% B in 32 min) gave PA-SiR-SNAP (3.0 mg, 49%) as a light green solid.

^{1}H NMR (400 MHz, CD_3OD): δ 8.32 (s, 1H), 7.47 – 7.58 (m, 4H), 7.36 – 7.42 (m, 3H), 7.25 – 7.30 (m, 3H), 5.95 (t, J = 7.2 Hz, 1H), 5.60 (s, 2H), 4.35 (s, 2H), 3.21 (s, 6H), 3.17 (s, 6H), 2.73 (q, J = 7.2 Hz, 2H), 2.41 (t, J = 7.2 Hz, 2H), 0.46 (s, 6H); ^{13}C NMR (101 MHz, CD_3OD): δ 174.9, 162.2 (d, J = 36.2 Hz), 161.1, 158.6, 153.8, 148.3, 146.1, 144.6, 143.2, 141.5, 140.8, 140.4, 139.6, 138.1, 135.4, 132.4, 131.1, 130.1, 128.7, 128.3, 122.4, 121.8, 120.5, 118.2, 117.8 (d, J = 290.6 Hz), 108.7, 70.6, 45.4, 44.3, 43.7, 37.0, 27.6, –2.6; ^{19}F NMR (376 MHz, CD_3OD): δ –77.14; HRMS (*m/z*): $[M + H]^+$ calcd. for $C_{37}H_{43}N_8O_2Si^+$, 647.3278; found, 647.3274.

10-(3-((2-(2-((6-Chlorohexyl)oxy)ethoxy)ethyl)amino)-3-oxopropylidene)-7-(dimethylamino)-*N*,*N*,5,5-tetramethyl-5,10-dihydrodibenzo[*b*,*e*]silin-3-aminium trifluoroacetate PA-SiR-C3-Halo (29)

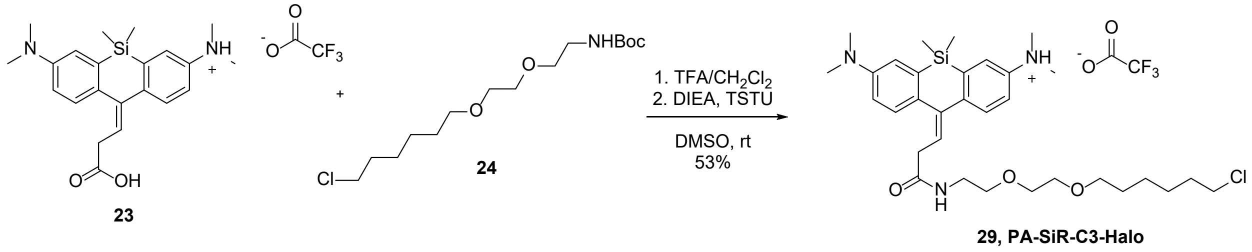

Supplementary Figure 29. Synthetic route. Reaction to give PA-SiR-C3-Halo.

A solution of **23** (4.0 mg, 8.1 µmol, 1.0 eq.) in DMSO (250 µL) was treated with DIEA (5.2 µL, 31.5 µmol, 4.0 eq.) and TSTU (3.8 mg, 12.6 µmol, 1.6 eq.). The mixture was shaken for 20 min at room temperature. In a separate vial a solution of Halo-NHBoc **24** (4.8 mg, 14.7 µmol, 1.8 eq.) in TFA/CH_2Cl_2 (2:8, 200 µL) was shaken for 5 min. The solution was evaporated and dried on the high vacuum for 1 h. The residue was taken up in DMSO (150 µL) and added to the other mixture. The mixture was shaken for 10 min and then acidified with TFA (15 µL). RP-HPLC (4 mL min^{-1}, 20% to 100% B in 32 min) gave PA-SiR-C3-Halo (3.0 mg, 53%) as a light blue solid.

^{1}H NMR (400 MHz, CD_3OD): δ 7.70 (d, *J* = 8.5 Hz, 1H), 7.59 (d, *J* = 2.6 Hz, 1H), 7.55 (d, *J* = 8.5 Hz, 1H), 7.50 – 7.41 (m, 2H), 7.24 (dd, *J* = 8.5, 2.7 Hz, 1H), 6.13 (t, *J* = 7.6 Hz, 1H), 3.64 – 3.48 (m, 6H), 3.52 (t, J = 6.6 Hz, 2H), 3.46 (t, J = 6.5 Hz, 2H), 3.39 (t, J = 5.4 Hz, 2H), 3.30 (d, J = 3.3 Hz, 2H), 3.24 (s, 6H), 3.16 (s, 6H), 1.72 (dq, J = 7.9, 6.7 Hz, 2H), 1.55 (tt, J = 7.5, 6.4 Hz, 2H), 1.49 – 1.22 (m, 4H), 0.49 (s, 6H); ^{13}C NMR (101 MHz, CD_3OD): δ 173.7, 161.9 (q, J = 36.5 Hz), 149.0, 147.0, 144.2, 143.2, 139.3, 138.7, 138.5, 131.0, 128.5, 126.3, 122.8, 121.2, 120.8, 117.7, 117.7 (q, J = 290.2 Hz), 72.2, 71.2, 71.2, 70.5, 45.8, 45.7, 43.8, 40.5, 38.1, 33.7, 30.5, 27.7, 26.4, –3.5; ^{19}F NMR (376 MHz, CD_3OD): δ –77.2; HRMS (m/z): $[M + H]^+$ calcd. for $C_{32}H_{49}ClN_3O_3Si^+$, 586.3226; found, 586.3226.

3,3'-(Diisopropylsilanediyl)bis(*N*,*N*-dimethylaniline) 31

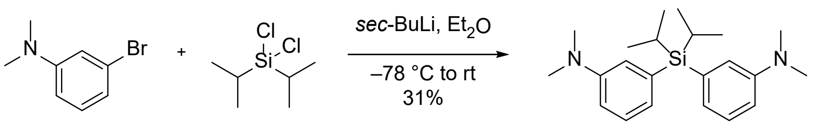

Supplementary Figure 30. Synthetic route. Reaction to give compound **31**.

Following general procedure **A**, flash column chromatography (SiO_2, hexane/EtOAc 100:0 → 80:20) gave **31** (0.440 g, 31%) as a colorless oil.

^{1}H NMR (400 MHz, $CDCl_3$): δ 7.21 – 7.29 (m, 2H), 6.90 – 7.00 (m, 4H), 6.81 (ddd, J = 8.3, 2.7, 1.0 Hz, 2H), 2.92 (s, 12H), 1.56 (hept, J = 7.3 Hz, 2H), 0.97 (d, J = 7.4 Hz, 12H); ^{13}C NMR (101 MHz, $CDCl_3$): δ 149.7, 133.9, 128.1, 125.2, 120.9, 113.7, 41.0, 17.8, 10.0; HRMS (*m/z*): $[M + H]^+$ calcd. for $C_{22}H_{35}N_2Si^+$, 355.2564; found, 355.2567.

3,3'-(Diisopropylsilanediyl)bis(4-bromo-*N*,*N*-dimethylaniline) 32

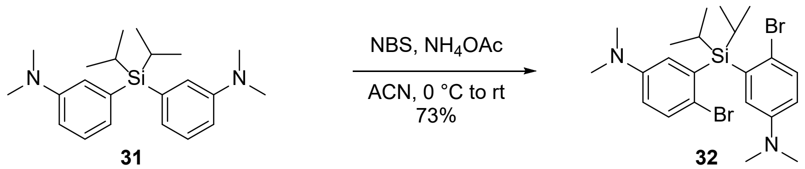

Supplementary Figure 31. Synthetic route. Reaction to give compound **32**.

Following general procedure **B**, flash column chromatography (SiO_2, hexane/CH_2Cl_2 100:0 → 0:100) gave **32** (1.087 g, 73%) as a white solid.

^{1}H NMR (400 MHz, $CDCl_3$): δ 7.33 (d, J = 8.7 Hz, 2H), 6.97 (d, J = 3.2 Hz, 2H), 6.60 (dd, J = 8.8, 3.3 Hz, 2H), 2.92 (s, 12H), 2.00 (p, J = 7.4 Hz, 2H), 1.17 (d, J = 7.5 Hz, 12H); ^{13}C NMR (101 MHz, $CDCl_3$): δ 148.6, 136.8, 133.4, 122.8, 117.6, 115.1, 40.8, 19.1, 12.7; HRMS (*m/z*): $[M + H]^+$ calcd. for $C_{22}H_{33}Br_2N_2Si^+$, 511.0774; found, 511.0779.

10-(3-Carboxypropylidene)-7-(dimethylamino)-5,5-diisopropyl-*N*,*N*-dimethyl-5,10-dihydrodibenzo[*b*,*e*]silin-3-aminium trifluoroacetate 33

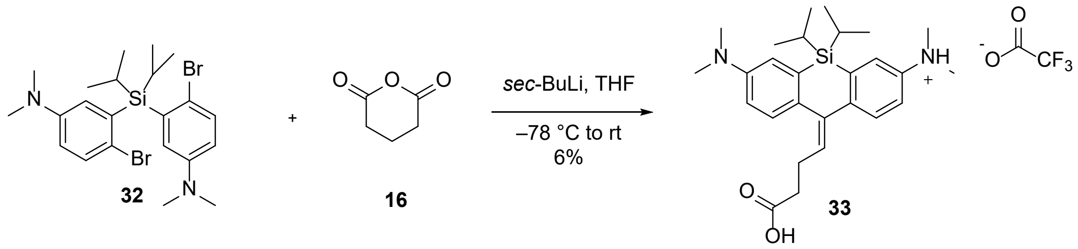

Supplementary Figure 32. Synthetic route. Reaction to give compound **33**.

Following general procedure **C**, flash column chromatography (SiO_2, hexane/EtOAc 100:0 → 50:50) and RP-HPLC (2 mL min^{-1}, 10% to 90% B in 32 min) gave **33** (6.7 mg, 6%) as a light blue solid.

^{1}H NMR (400 MHz, CD_3OD): δ 7.69 (d, J = 8.5 Hz, 1H), 7.48 – 7.61 (m, 4H), 7.37 (dd, J = 8.6, 2.7 Hz, 1H), 5.90 (t, J = 7.2 Hz, 1H), 3.24 (s, 6H), 3.20 (s, 6H), 2.65 (q, J = 7.6 Hz, 2H), 2.41 (t, J = 7.4 Hz, 2H), 1.57 (br. s, 2H), 1.09 (br. s, 12H); ^{13}C NMR (101 MHz, CD_3OD): δ 176.5, 161.9 (q, J = 36.2 Hz), 150.5, 145.7, 143.9, 142.2, 141.7, 136.0, 134.7, 133.7, 131.6, 128.7, 123.9, 123.0, 121.0, 116.3 (q, J = 292.8 Hz), 45.8, 44.5, 34.8, 26.9, 18.4, 12.9; ^{19}F NMR (376 MHz, CD_3OD): δ –77.16; HRMS (*m/z*): [M – H]$^-$ calcd. for $C_{27}H_{37}N_2O_2Si^-$, 449.2630; found, 449.2622.

5-Bromo-2-fluoro-*N*,*N*-dimethylaniline 35

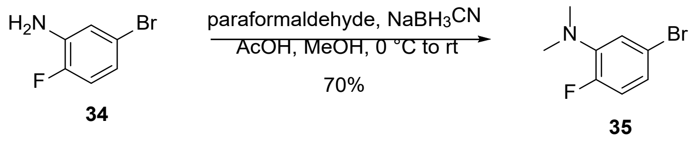

Supplementary Figure 33. Synthetic route. Reaction to give compound **35**.

A solution of 5-bromo-2-fluoroaniline **34** (5.0 g, 26.3 mmol, 1.0 eq.) in MeOH (30 mL) was treated with acetic acid (40 mL) and paraformaldehyde (3.9 g, 131.6 mmol, 5.0 eq.). The mixture was cooled down to 0 °C and stirred for 15 min. $NaBH_3CN$ (5.0 g, 78.9 mmol, 3.0 eq.) was added portion wise to the mixture over 10 min. The mixture was warmed up to room temperature and was stirred for 16 h. The mixture was evaporated and then neutralized with aqueous NaOH

solution (4 mL, 5 M). The aqueous layer was extracted with CH_2Cl_2 (3 x 100 mL). The combined organic layers were dried over $MgSO_4$, filtered and evaporated to afford the crude product. Flash column chromatography (SiO_2, hexane/EtOAc 100:0 → 85:15) gave **35** (3.990 g, 70%) as a yellow oil.

^{1}H NMR (400 MHz, $CDCl_3$): δ 6.82 – 6.99 (m, 3H), 2.85 (d, J = 1.0 Hz, 6H); ^{13}C NMR (101 MHz, $CDCl_3$): δ 154.1 (d, J = 245.3 Hz), 142.1 (d, J = 9.7 Hz), 123.3 (d, J = 7.9 Hz), 121.2 (d, J = 3.9 Hz), 117.6 (d, J = 22.8 Hz), 116.9 (d, J = 3.3 Hz), 42.7 (d, J = 4.5 Hz); ^{19}F NMR (376 MHz, $CDCl_3$): δ –124.69 – –124.58 (m); HRMS (*m/z*): [M + H]$^+$ calcd. for $C_8H_{10}BrFN^+$, 217.9975; found, 217.9978.

5,5'-(Dimethylsilanediyl)bis(2-fluoro-*N*,*N*-dimethylaniline) 36

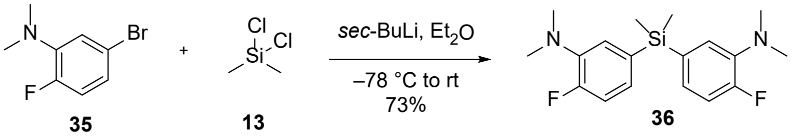

Supplementary Figure 34. Synthetic route. Reaction to give compound **36**.

Following general procedure **A**, flash column chromatography (SiO_2, hexane/EtOAc 100:0 → 85:15) gave **36** (2.110 g, 73%) as a yellow oil.

^{1}H NMR (400 MHz, $CDCl_3$): δ 6.98 – 7.06 (m, 6H), 2.83 (d, J = 0.9 Hz, 12H), 0.52 (s, 6H); ^{13}C NMR (101 MHz, $CDCl_3$): δ 156.3 (d, J = 248.0 Hz), 140.3 (d, J = 8.0 Hz), 134.0 (d, J = 4.3 Hz), 127.6 (d, J = 7.5 Hz), 124.0 (d, J = 3.4 Hz), 115.9 (d, J = 19.8 Hz), 43.0 (d, J = 3.9 Hz), –1.9; ^{19}F NMR (376 MHz, $CDCl_3$): δ –121.34 – –121.21 (m); HRMS (*m/z*): [M + H]$^+$ calcd. for $C_{18}H_{25}F_2N_2Si^+$, 335.1750; found, 335.1754.

5,5'-(Dimethylsilanediyl)bis(4-bromo-2-fluoro-*N*,*N*-dimethylaniline) 37

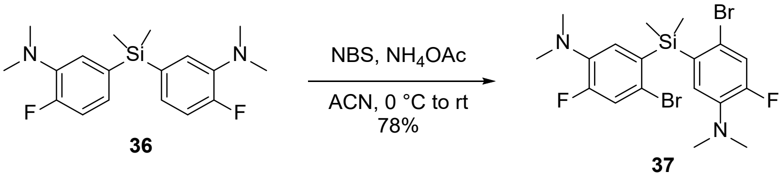

Supplementary Figure 35. Synthetic route. Reaction to give compound **37**.

Following general procedure **B**, flash column chromatography (SiO_2, hexane/CH_2Cl_2 100:0 → 0:100) gave **37** (2.329 g, 78%) as a beige solid.

^{1}H NMR (400 MHz, $CDCl_3$): δ 7.20 (d, J = 12.5 Hz, 2H), 6.94 (d, J = 10.2 Hz, 2H), 2.80 (d, J = 1.0 Hz, 12H), 0.74 (s, 6H); ^{13}C NMR (101 MHz, $CDCl_3$): δ 155.6 (d, J = 252.7 Hz), 139.4 (d, J = 7.5 Hz), 134.1 (d, J = 4.0 Hz), 126.7 (d, J = 4.0 Hz), 120.9 (d, J = 23.2 Hz), 119.9 (d, J = 8.6 Hz), 42.7 (d, J = 4.0 Hz), –0.8; ^{19}F NMR (376 MHz, $CDCl_3$): δ –118.89 (t, J = 11.3 Hz); HRMS (*m/z*): $[M + H]^+$ calcd. for $C_{18}H_{23}Br_2F_2N_2Si^+$, 490.9960; found, 490.9954.

10-(3-Carboxypropylidene)-7-(dimethylamino)-2,8-difluoro-*N*,*N*,5,5-tetramethyl-5,10-dihydrodibenzo[*b*,*e*]silin-3-aminium trifluoroacetate 38

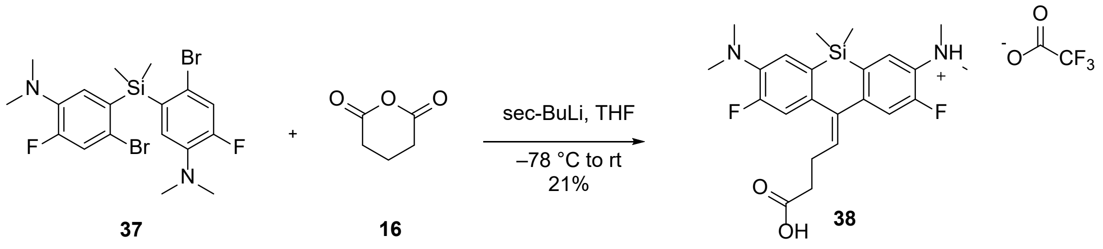

Supplementary Figure 36. Synthetic route. Reaction to give compound **38**.

Following general procedure **C**, flash column chromatography (SiO_2, hexane/EtOAc 80:20 → 0:100) and RP-HPLC (3 mL min^{-1}, 10% to 90% B in 32 min) gave **38** (24 mg, 21%) as a white solid.

^{1}H NMR (400 MHz, CD_3OD): δ 7.58 (d, J = 9.0 Hz, 1H), 7.54 (d, J = 9.3 Hz, 1H), 7.40 (d, J = 13.3 Hz, 1H), 7.35 (d, J = 13.6 Hz, 1H), 6.00 (t, J = 7.3 Hz, 1H), 3.15 (s, 6H), 3.10 (s, 6H), 2.69 (q, J = 7.2 Hz, 2H), 2.47 (t, J = 7.1 Hz, 2H), 0.48 (s, 6H); ^{13}C NMR (101 MHz, CD_3OD): δ 176.4, 163.2 (d, J = 34.8 Hz), 161.1 (q, J = 38.1 Hz), 157.8 (d, J = 85.7 Hz), 155.3 (d, J = 84.7 Hz), 150.0 (d, J = 7.1 Hz), 142.5 (d, J = 5.5 Hz), 139.9, 135.7 (d, J = 8.2 Hz), 134.9 (d, J = 3.5 Hz), 134.5, 133.7 (d, J = 11.7 Hz), 133.3 (d, J = 3.9 Hz), 124.9, 118.0 (d, J = 20.3 Hz), 117.2 (q, J = 288.5 Hz), 115.3 (d, J = 20.0 Hz), 45.3 (d, J = 2.5 Hz), 44.7 (d, J = 2.9 Hz), 34.7, 26.5, –3.4; ^{19}F NMR (376 MHz, CD_3OD): δ –77.45, –123.44 (dd, J = 13.3, 9.3 Hz); HRMS (*m/z*): $[M – H]^–$ calcd. for $C_{23}H_{27}F_2N_2O_2Si^–$, 429.1815; found, 429.1816.

Protein amino acid sequence of HaloTag

MASWSHPQFEKGADDDDKVPHGSEIGTGFPFDPHYVEVLGERMHYVDVGPRDGTPVLFLHGNPTSSYVW
RNIIPHVAPTHRCIAPDLIGMGKSDKPDLGYFFDDHVRFMDAFIEALGLEEVVLVIHDWGSALGFHWAKRNP
ERVKGIAFMEFIRPIPTWDEWPEFARETFQAFRTTDVGRKLIIDQNVFIEGTLPMGVVRPLTEVEMDHYREPF
LNPVDREPLWRFPNELPIAGEPANIVALVEEYMDWLHQSPVPKLLFWGTPGVLIPPAEAARLAKSLPNCKAV
DIGPGLNLLQEDNPDLIGSEIARWLSTLEISGAPGFSSISAHHHHHHHHHH

Green: Strep-tag, Red: HaloTag, Purple: His-tag

Protein amino acid sequence of SNAP-tag

MASWSHPQFEKGADDDDKVPHMDKDCEMKRTTLDSPLGKLELSGCEQGLHEIIFLGKGTSAADAVEVPAP
AAVLGGPEPLMQATAWLNAYFHQPEAIEEFPVPALHHPVFQQESFTRQVLWKLLKVVKFGEVISYSHLAALA
GNPAATAAVKTALSGNPVPILIPCHRVVQGDLDVGGYEGGLAVKEWLLAHEGHRLGKPGLGAPGFSSISAH
HHHHHHHH

Green: Strep-tag, Red: SNAP-tag, Purple: His-tag

Protein amino acid sequence of mEOS3.2:HaloTag

MASWSHPQFEKGADDDDKVPHMSAIKPDMKIKLRMEGNVNGHHFVIDGDGTGKPFEGKQSMDLEVKEG
GPLPFAFDILTTAFHYGNRVFAKYPDNIQDYFKQSFPKGYSWERSLTFEDGGICNARNDITMEGDTFYNKVRF
YGTNFPANGPVMQKKTLKWEPSTEKMYVRDGVLTGDIEMALLLEGNAHYRCDFRTTYKAKEKGVKLPGAH
FVDHCIEILSHDKDYNKVKLYEHAVAHSGLPDNARRGRLEVLFQGPKAFLEGSEIGTGFPFDPHYVEVLGERM
HYVDVGPRDGTPVLFLHGNPTSSYVWRNIIPHVAPTHRCIAPDLIGMGKSDKPDLGYFFDDHVRFMDAFIEA
LGLEEVVLVIHDWGSALGFHWAKRNPERVKGIAFMEFIRPIPTWDEWPEFARETFQAFRTTDVGRKLIIDQN
VFIEGTLPMGVVRPLTEVEMDHYREPFLNPVDREPLWRFPNELPIAGEPANIVALVEEYMDWLHQSPVPKLL
FWGTPGVLIPPAEAARLAKSLPNCKAVDIGPGLNLLQEDNPDLIGSEIARWLSTLEISGAPGFSSISAHHHHHH
HHHH

Green: Strep-tag, Red: HaloTag, Blue: mEOS3.2, Purple: His-tag

Protein amino acid sequence of SNAP-tag:EGFP:HaloTag

MASWSHPQFEKGADDDDKVPHMDKDCEMKRTTLDSPLGKLELSGCEQGLHEIIFLGKGTSAADAVEVPAP
AAVLGGPEPLMQATAWLNAYFHQPEAIEEFPVPALHHPVFQQESFTRQVLWKLLKVVKFGEVISYSHLAALA

GNPAATAAVKTALSGNPVPILIPCHRVVQGDLDVGGYEGGLAVKEWLLAHEGHRLGKPGLGGRLEVLFQGP
KAFLEMVSKGEELFTGVVPILVELDGDVNGHKFSVSGEGEGDATYGKLTLKFICTTGKLPVPWPTLVTTLTYGV
QCFSRYPDHMKQHDFFKSAMPEGYVQERTIFFKDDGNYKTRAEVKFEGDTLVNRIELKGIDFKEDGNILGHK
LEYNYNSHNVYIMADKQKNGIKVNFKIRHNIEDGSVQLADHYQQNTPIGDGPVLLPDNHYLSTQSALSKDPN
EKRDHMVLLEFVTAAGITLGMDELYKIGTGFPFDPHYVEVLGERMHYVDVGPRDGTPVLFLHGNPTSSYVW
RNIIPHVAPTHRCIAPDLIGMGKSDKPDLGYFFDDHVRFMDAFIEALGLEEVVLVIHDWGSALGFHWAKRNP
ERVKGIAFMEFIRPIPTWDEWPEFARETFQAFRTTDVGRKLIIDQNVFIEGTLPMGVVRPLTEVEMDHYREPF
LNPVDREPLWRFPNELPIAGEPANIVALVEEYMDWLHQSPVPKLLFWGTPGVLIPPAEAARLAKSLPNCKAV
DIGPGLNLLQEDNPDLIGSEIARWLSTLEISGAPGFSSISAHHHHHHHHHH

Green: Strep-tag, Red: SNAP-tag, Brown: EGFP, Blue: HaloTag, Purple: His-tag

Supplementary References

1 van Walree, Cornelis A. *et al.* Charge-Transfer Interactions in 4-Donor 4′-Acceptor Substituted 1,1-Diphenylethenes. *European Journal of Organic Chemistry* **2004**, 3046-3056, (2004).

2 Matsui, M., Tsuge, M., Shibata, K. & Muramatsu, H. Photochromism of 1,1-Diaryl-1-alkanols. *Bulletin of the Chemical Society of Japan* **67**, 1753-1755, (1994).

3 Aaron, C. & Barker, C. C. 495. Steric effects in di- and tri-arylmethanes. Part VIII. Electronic absorption spectra of planar derivatives of Michler's Hydrol Blue. *Journal of the Chemical Society (Resumed)*, 2655-2662, (1963).

4 Amat-Guerri, F., Martin, M. E., Martinez-Utrilla, R. t. l. & Pascual, C. Structures of Molecular and Ionic Forms of Succinylfluorescein in Solution and in the Solid State. *J. Chem. Research(S)*, 184-185, (1988).

5 Dodiuk, H. & Kosower, E. M. Multiple fluorescences from the excited state of N-methyl-2-N-phenyl-amino-6-naphthalenesulphonate in glycerol: Fast proton transfer. *Chemical Physics Letters* **34**, 253-257, (1975).

6 Kosower, E. M. & Dodiuk, H. Multiple fluorescences. II. A new scheme for 4-(N,N-dimethylamino)benzonitrile including proton transfer. *Journal of the American Chemical Society* **98**, 924-929, (1976).

7 Grimm, J. B. *et al.* Bright photoactivatable fluorophores for single-molecule imaging. *Nature Methods* **13**, 985-988, (2016).

8 Marsh, R. J. *et al.* Artifact-free high-density localization microscopy analysis. *Nature Methods* **15**, 689-692, (2018).

9 Ovesný, M., Křížek, P., Borkovec, J., Švindrych, Z. & Hagen, G. M. ThunderSTORM: a comprehensive ImageJ plug-in for PALM and STORM data analysis and super-resolution imaging. *Bioinformatics* **30**, 2389-2390, (2014).

10 Lukinavičius, G. *et al.* A near-infrared fluorophore for live-cell super-resolution microscopy of cellular proteins. *Nature Chemistry* **5**, 132-139, (2013).

11 Halabi, E. A., Pinotsi, D. & Rivera-Fuentes, P. Photoregulated fluxional fluorophores for live-cell super-resolution microscopy with no apparent photobleaching. *Nature Communications* **10**, 1232, (2019).

12 Habuchi, S., Tsutsui, H., Kochaniak, A. B., Miyawaki, A. & van Oijen, A. M. mKikGR, a Monomeric Photoswitchable Fluorescent Protein. *PLOS ONE* **3**, e3944, (2008).

13 Tsutsui, H., Karasawa, S., Shimizu, H., Nukina, N. & Miyawaki, A. Semi-rational engineering of a coral fluorescent protein into an efficient highlighter. *EMBO reports* **6**, 233-238, (2005).

14 Keppler, A. *et al.* A general method for the covalent labeling of fusion proteins with small molecules in vivo. *Nature Biotechnology* **21**, 86-89, (2002).

15 Los, G. V. *et al.* HaloTag: A Novel Protein Labeling Technology for Cell Imaging and Protein Analysis. *ACS Chemical Biology* **3**, 373-382, (2008).

16 Grimm, J. B., Brown, T. A., Tkachuk, A. N. & Lavis, L. D. General Synthetic Method for Si-Fluoresceins and Si-Rhodamines. *ACS Central Science* **3**, 975-985, (2017).

17 Fischer, C. & Sparr, C. Direct Transformation of Esters into Heterocyclic Fluorophores. *Angewandte Chemie International Edition* **57**, 2436-2440, (2018).
