# Supplementary material for: Photoactivation of silicon rhodamines via a light-induced protonation
Source: Nat Commun. 2019 Oct 8;10:4580. doi: 10.1038/s41467-019-12480-3 (PMC6783549; doi:10.1038/s41467-019-12480-3)
Supplement: Supplementary file 3 — Description of Additional Supplementary Files [file 41467_2019_12480_MOESM3_ESM.pdf]

Description of Additional Supplementary Files

File name: Supplementary Movie 1

Description: SMLM rolling frame movie of mitochondria dynamics. U-2 OS cells expressing TOMM20-Halo labeled with PA-SiR-Halo (0.5 µM, 1 h). Each frame is reconstructed from 200 frames (10 s). Scale bar 1 µm.
