# Supplementary material for: Photoactivation of silicon rhodamines via a light-induced protonation
Source: Nat Commun. 2019 Oct 8;10:4580. doi: 10.1038/s41467-019-12480-3 (PMC6783549; doi:10.1038/s41467-019-12480-3)
Supplement: Supplementary file 5 — Reporting Summary [file 41467_2019_12480_MOESM5_ESM.pdf]

nature research

Corresponding author(s): Luc Reymond and Kai Johnsson

Last updated by author(s): Jul 26, 2019

Reporting Summary

Nature Research wishes to improve the reproducibility of the work that we publish. This form provides structure for consistency and transparency in reporting. For further information on Nature Research policies, see Authors & Referees and the Editorial Policy Checklist.

Statistics

For all statistical analyses, confirm that the following items are present in the figure legend, table legend, main text, or Methods section.

n/a	Confirmed	
☐	☒	The exact sample size (*n*) for each experimental group/condition, given as a discrete number and unit of measurement
☐	☒	A statement on whether measurements were taken from distinct samples or whether the same sample was measured repeatedly
☒	☐	The statistical test(s) used AND whether they are one- or two-sided<br>*Only common tests should be described solely by name; describe more complex techniques in the Methods section.*
☒	☐	A description of all covariates tested
☐	☒	A description of any assumptions or corrections, such as tests of normality and adjustment for multiple comparisons
☐	☒	A full description of the statistical parameters including central tendency (e.g. means) or other basic estimates (e.g. regression coefficient) AND variation (e.g. standard deviation) or associated estimates of uncertainty (e.g. confidence intervals)
☒	☐	For null hypothesis testing, the test statistic (e.g. *F*, *t*, *r*) with confidence intervals, effect sizes, degrees of freedom and *P* value noted<br>*Give P values as exact values whenever suitable.*
☒	☐	For Bayesian analysis, information on the choice of priors and Markov chain Monte Carlo settings
☒	☐	For hierarchical and complex designs, identification of the appropriate level for tests and full reporting of outcomes
☐	☒	Estimates of effect sizes (e.g. Cohen's *d*, Pearson's *r*), indicating how they were calculated

Our web collection on statistics for biologists contains articles on many of the points above.

Software and code

Policy information about availability of computer code

Data collection	NMR: Bruker TopSpin 3.5 and Bruker TopSpin 3.6<br>HRMS: Bruker otofControl 4.1, Bruker Hystar 4.1 SR2 software or Waters MassLynx MS software<br>LC-MS: Shimadzu LabSolutions 5.89<br>UV-Vis: JASCO Spectra Manger 2.13.00<br>Flourimeter and Plate reader: Tecan Sparkcontrol Method Editor V.2.2, JASCO Spectra Manager 2.14.02<br>Quantaurus: Hamamatsu Quantaurus - QY Plus 4.2.0<br>Structure optimization and HOMO/LUMO calculations: Gaussian 09<br>Microscopy: Leica LAS X 1.9.0.13747 (GSD), Leica LAS X 3.6.0.20104 (Widefield), Leica LAS X 3.1.1.15751 (Confocal), µManager 1.4.22 (Custom)<br>X-ray crystallography: CrysAlisPro 1.171.40.53a, SIR2019 19.03, SHELXL Version 2018/3
Data analysis	Origin Pro 2018b b9.5.5.409, Image J 1.52i + Plugins (ThunderSTORM, TrackMate and HAWK detailed in the SI), MATLAB R2017b 9.3.0.713579, custom code written in MATLAB (SMAP) deposited on git (github.com/jries/SMAP), Avogadro 1.2.0, Bruker DataAnalysis 4.4 SR1, MestReNova 12.0.3-21384.<br>X-ray crystallography: CrysAlisPro 1.171.40.53a, SIR2019 19.03, SHELXL Version 2018/3

For manuscripts utilizing custom algorithms or software that are central to the research but not yet described in published literature, software must be made available to editors/reviewers. We strongly encourage code deposition in a community repository (e.g. GitHub). See the Nature Research guidelines for submitting code & software for further information.

Data

Policy information about availability of data

All manuscripts must include a data availability statement. This statement should provide the following information, where applicable:

- Accession codes, unique identifiers, or web links for publicly available datasets
- A list of figures that have associated raw data
- A description of any restrictions on data availability

The data supporting the findings of this study are available within the paper and its Supplementary Information and are available from the corresponding author upon reasonable request

Field-specific reporting

Please select the one below that is the best fit for your research. If you are not sure, read the appropriate sections before making your selection.

☒ Life sciences ☐ Behavioural & social sciences ☐ Ecological, evolutionary & environmental sciences

For a reference copy of the document with all sections, see nature.com/documents/nr-reporting-summary-flat.pdf

Life sciences study design

All studies must disclose on these points even when the disclosure is negative.

Sample size	Sample size was based on experience in prior studies and sized to allow significance in biologically relevant effect sizes.
Data exclusions	No data was excluded
Replication	In vitro measurements were performed in triplicates. Microscopy experiments were performed on different sample preparations and different field of views. All replicates were successful.
Randomization	No randomization was applied
Blinding	No blinding was applied

Reporting for specific materials, systems and methods

We require information from authors about some types of materials, experimental systems and methods used in many studies. Here, indicate whether each material, system or method listed is relevant to your study. If you are not sure if a list item applies to your research, read the appropriate section before selecting a response.

Materials & experimental systems

n/a	Involved in the study	
☒	☐	Antibodies
☐	☒	Eukaryotic cell lines
☒	☐	Palaeontology
☒	☐	Animals and other organisms
☒	☐	Human research participants
☒	☐	Clinical data

Methods

n/a	Involved in the study	
☒	☐	ChIP-seq
☒	☐	Flow cytometry
☒	☐	MRI-based neuroimaging

Eukaryotic cell lines

Policy information about cell lines

Cell line source(s)	U-2 OS provided by ATCC HTB-96.<br>U-2 OS FlpIn cell line was generated based on reported work (Molecular and Cellular Biology 2006, 26 (12), 4642-4651) and described in the method in supporting information.<br>HeLa provided by DSMZ ACC 57.<br>COS-7 provided by ATCC CRL-1651.<br>U-2 OS provided also by ATCC-HTB-96, for establishment of endogenously tagged NUP96-Halo cell line.
Authentication	Cell lines were not further authenticated.
Mycoplasma contamination	Cell lines have been tested and are negative.

Commonly misidentified lines<br>(See ICLAC register)	Not applicable as no commonly misidentified cell lines were used.
